# Taxon‐rich phylogeny and taxonomy of the genus *Phacus* (Euglenida) based on morphological and molecular data

**DOI:** 10.1111/jpy.13028

**Published:** 2020-06-26

**Authors:** Maja Łukomska‐Kowalczyk, Alicja Fells, Katarzyna Chaber, Rafał Milanowski, Bożena Zakryś

**Affiliations:** ^1^ Institute of Evolutionary Biology Faculty of Biology, Biological and Chemical Research Center University of Warsaw ul. Żwirki i Wigury 101 02‐089 Warszawa Poland

**Keywords:** Euglenida, environmental sampling, nSSU rDNA, *Phacus*, phylogeny, taxonomical revision

## Abstract

Morphological and molecular features were analyzed for a species of *Phacus* to better understand the phylogenetic relationships among them and establish the taxonomy. Phylogenetic analyses were based on nSSU rDNA and the research resulted in 55 new sequences. The study included species available in algal collections and those isolated directly from the environment in Poland and the Czech Republic. As a result, the obtained phylogenetic tree of *Phacus* includes 50 species, out of which 7 are represented on a tree for the first time (*Phacus anacoelus, P. anomalus*,* P. curvicauda*,* P. elegans*,* P. lismorensis*,* P. minutus* and *P. stokesii*) and many have been taxonomically verified. For all verified species, diagnostic descriptions were amended, the naming was reordered and epitypes were designated.

**Abbreviations**
BI: Bayesian inference
rbs: rapid bootstrap
ML: maximum likelihood
nt: nucleotide
pp: posterior probability

*Phacus* was described in the 19^th^ century (Dujardin 1841) and currently includes approximately 300 species names, of which 174 are taxonomically accepted (http://www.algaebase.org; Guiry and Guiry 2020). The number of species changes as taxonomic verifications (based on morphological and DNA sequence data) are ongoing (Kosmala et al. 2007, Karnkowska‐Ishikawa et al. 2010, Kim et al. 2010, Linton et al. 2010, Bennett and Triemer 2012, Kim and Shin 2014, Kim et al. 2015, Łukomska‐Kowalczyk et al. 2015). As a result, our understanding of the phylogenetic relationships among them is also constantly changing. The research cited above did not validate *Phacus*' separation into two subgenera, *Chlorophacus* (green forms) and *Hyalophacus* (colorless) as suggested by Pochmann (1942), or into four sections for the green forms (Proterophacus – cells flattened and leaf‐shaped; Pleuraspis – spherical or flat with ribbing instead of striae; Acanthochloris – slightly flattened with papillae on the surface; Kampylopter – round, triangular in cross‐section). First, it was shown that species from the Pleuraspis section are in fact representatives of *Monomorphina* (Marin et al. 2003). Later, when both *Lepocinclis salina* and *Euglena limnophila*, neither of which have flattened cells, were included in *Phacus*, the description of the genus underwent changes (Linton et al. 2010). Recently, *Phacus horridus* (=*P. hispidulus* from the Acanthochloris section) was reclassified as a member of *Lepocinclis* (Bennett and Triemer 2012, Łukomska‐Kowalczyk et al. 2020). As a result of all previous morphological‐molecular studies, descriptions were emended, epitypes were designated, many species were renamed, and nine new taxa were described (Kim et al. 2010, Łukomska‐Kowalczyk et al. 2015). Currently, *Phacus* is characterized by semi‐rigid to rigid cells, usually laterally compressed (with the exception of *P. limnophilus* and *P. salinus*), in most cases leaf‐shaped, sometimes twisted. They possess numerous chloroplasts of uniform shape (numerous, small, disc‐shaped, parietal, without pyrenoids). All species (with the exception of *P. salinus*) have dimorphic in size paramylon grains – in most cases the large grains are plate‐ or ring‐shaped. Colourless forms are known (e.g., *Phacus ocellatus*; Marin et al. 2003). *Phacus* is currently classified in the Phacaceae family, together with the representatives of the *Lepocinclis* and *Discoplastis* genera (Kim et al. 2010).

The most recently published phylogenetic trees of *Phacus* include 43 species (Kim et al. 2015, Łukomska‐Kowalczyk et al. 2015), some of which have been misidentified. We conducted this study to increase the number of species and strains representing *Phacus* in the phylogenetic trees and to perform comparative morphological and DNA sequence studies on new strains (=isolates). This will allow an estimation of morphological and genetic diversity and verification of morphological diagnostic features for particular taxa (well‐established clades) on a molecular phylogenetic tree; (b) the reconstruction of phylogenetic relationships and (c) taxonomic verification, amending diagnoses and designating epitypes for well‐distinguished taxa.

## MATERIAL AND METHODS

### Sampling and morphological study

**Fig. 1:**
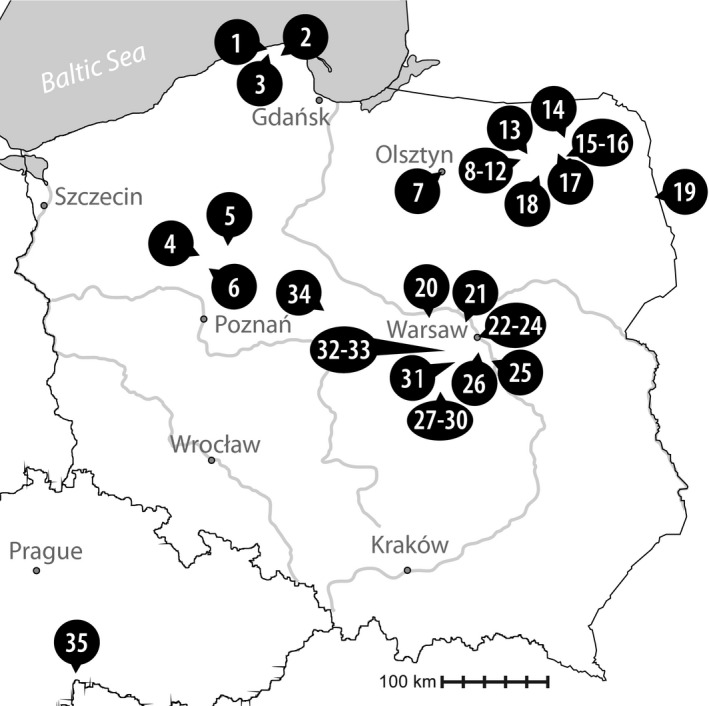
Map illustrating sampling locations in Poland and the Czech Republic. The names of lakes or towns in which the studied water bodies were located are marked with numbers: (1) Kopalino; (2) Strzebielinek; (3) Choczewo; (4) Chodzież; (5) Sokolec; (6) Budzyń 1 and 2; (7) Olsztyn; (8) Urwitałt 10; (9) Urwitałt 15; (10) Urwitałt 17; (11) Urwitałt 19; (12) Urwitałt 20; (13) Grabówek; (14) Doliwy; (15) Oracze; (16) Woszczele; (17) Jeziorowskie; (18) Pilchy; (19) Wojnowce; (20) Oracze; (21) Górki; (22) Łazienki; (23) Jelonki; (24) Żerań; (25) Baniocha; (26) Zgorzała; (27) Biała Rawska; (28) Regnów; (29) Cielądz; (30) Ossowice; (31) Nowa Bukówka; (32) Izdebno Nowe; (33) Izdebno Kościelne 1 and 2; (34) Mąkolno (35) Lásenický Stav.

During nine seasons (2011–2019), plankton samples were collected from 37 eutrophic water bodies located in Poland and one located in the Czech Republic using a plankton net with a mesh size of 10 µm (Fig. 1). Samples were screened in terms of their species diversity, and then a number of cells (7–300; see Table S1) exhibiting the same morphology were isolated from the sample using a micromanipulator (MM‐89 Narishige) with a micropipette installed on a Nikon Ni‐U microscope (Nikon, Tokyo, Japan). Morphological studies (descriptions and measurements) and documentation (photographs and video clips) of the isolated cells (isolates) of the *Phacus* morphotypes and those from six strains from algae collections (ACOI 1753, ACOI 1754, ACOI 1755, ACOI 1757, ACOI 3237, AICB 324) were executed with a NIKON Eclipse E‐600 microscope with differential interference contrast, equipped with the NIS Elements Br 3.1 software (Nikon) for image processing and recording. Photographs (and video clips) were taken using a NIKON DX‐1200 digital camera connected to the microscope. The NIS Elements Br measurement program was used for morphometric studies; three parameters were measured for the cells of each isolate (strain) – cell length, cell width, and tail length (which was defined as the hyaline projection); measurements were conducted from photographs of the isolates (strains). The data was analysed using the R v.3.2.0 software (R Development Core Team, 2008); means and standard deviations are given in Table 1. The isolates (i.e., morphologically identical cells) were transferred through multiple drops of sterile media (to clean the sample), and kept frozen at −80°C until DNA extraction.

**Table 1:** Comparison of morphological traits among the study's isolates/strains of *Phacus*.

Taxon	Isolate/strain	Number of measured cells	Cell length (μm) Mean ± SD	Cell width (μm)\Mean ± SD	Tail length (μm) Mean ± SD	Cell shape	Tail turning
*P. acuminatus*	ACOI 1753	107	31.2 ± 1.2	19.5 ± 1.8	2.4 ± 0.4	Flat, leaf‐like, wide‐oval	Bent sideways
	ACOI 1754	97	29.6 ± 0.9	23.3 ± 1.7	2.2 ± 0.3		
	ACOI 3237	105	30.7 ± 1.2	22.8 ± 1.7	2.1 ± 0.4		
	UTEX 1317	107	31.2 ± 1.5	18.5 ± 1.6	2.7 ± 0.6		
	UW2269INo	28	31.3 ± 1.2	22.6 ± 1.0	2.3 ± 0.4		
*P. alatus*	UW1898NBu	43	42.8 ± 1.1	30.5 ± 1.4	6.5 ± 1.0	Thick, wide‐oval, slightly flattened	Bent dorsally
	UW2300Str	15	42.5 ± 1.5	29.4 ± 0.9	7.8 ± 0.9		
*P. anacoelus*	UW1960Reg	33	49.5 ± 2.4	34.9 ± 1.9	11.6 ± 1.7	Thick, spherical, slightly flattened	Bent dorsally
	UW2219IK1	30	47.4 ± 2.2	30.6 ± 2.5	10.9 ± 1.3		
*P. ankylonoton*	CCAC 0043	110	39.1 ± 3.1	24.6 ± 1.9	8.7 ± 1.7	Thick, oval, triangular when cross‐sectioned	Straight
*P. anomalus*	UW1842LSt	7	35.9 ± 1.2	32.4 ± 1.0	5.7 ± 1.2	Thick, trapezoid‐shaped, slightly flattened	Bent dorsally
	UW1936Wos	2	41.1 ± 3.2	27.9 ± 0.5	7.8 ± 0.4		
	UW1984Cie	26	34.6 ± 1.7	26.3 ± 1.6			
	UW2193BRa	42	42.9 ± 2.7	30.3 ± 1.7	6.7 ± 1.4		
*P. applanatus*	CCAC 2604 B	92	39.5 ± 2.8	20.1 ± 2.4	6.8 ± 1.1	Flat, elongated oval‐like	Straight
*P. arnoldii*	CCAC 2432 B	10	57.8 ± 1.9	29.0 ± 1.9	12.8 ± 1.2	Spherical, slightly twisted, triangular in cross section	Straight
	UW1650Ur10	15	63.0 ± 2.5	38.0 ± 1.4	16.0 ± 2.0		
	UW2313Pil	30	60.4 ± 3.4	34.3 ± 1.3	12.8 ± 1.1		
*P. caudatus*	AICB 324	111	34.0 ± 1.1	20.5 ± 1.6	3.5 ± 0.6	Flat, elongated oval‐like	Bent ventrally
	ASW 08020 (CCAC 2415 B)	108	39.3 ± 3.1	24.8 ± 2.2	8.4 ± 1.7		
	CCAC 0034	109	34.6 ± 2.0	15.9 ± 1.7	5.5 ± 1.0		
	UW2228INo	11	35.0 ± 1.6	17.2 ± 0.9	4.3 ± 0.8		
	UW2383Zer	19	37.8 ± 1.6	20.8 ± 1.2	4.4 ± 0.6		
*P. curvicauda*	UW2163Wos	2	28.5 ± 0.0	20.8 ± 0.5	2.7 ± 0.7	Thick, wide‐oval	Bent dorsally
	UW2262INo	12	32.4 ± 1.8	21.3 ± 1.5	3.8 ± 0.7		
	UW2461Mak	34	31.2 ± 1.25	24.48 ± 1.26	3.3 ± 0.43		
	UW2468Jel	57	32.3 ± 1.1	25.1 ± 1.3	3.5 ± 0.5		
*P. elegans*	UW1837Ols	1	142.3	39.0	51.2	Flat, elongated oval‐like	Straight
	UW2064Ur19	12	124.8 ± 4.9	47.4 ± 2.5	39.9 ± 3.6		
*P. gigas*	MSU	1	123	71	28	Flat, round	Bent sideways
	UW1669Ora	40	122.2 ± 2.5	76.7 ± 2.3	27.6 ± 2.0		
	UW1823Ur19	16	125.2 ± 4.0	81.2 ± 2.0	24.7 ± 2.7		
*P. hamatus*	CCAC 2605 B	1	52.3	28.9	10.3	Flat, spoon‐shaped, convex	Bent ventrally
	UW1900NBu	23	63.8 ± 1.7	39.1 ± 1.7	12.7 ± 1.5		
	UW1975Sok	4	53.2 ± 1.0	33.2 ± 1.5	9.9 ± 0.9		
*P. limnophilus*	ACOI 1026	1	62	8.5		Fusiform, not flattened	Straight
	UW1988Reg	60	80.4 ± 2.6	9.4 ± 1.2			
*P. lismorensis*	UW1665Ur10	2	124.5 ± 13.4	31.0 ± 1.4	45.5 ± 7.8	Flat, long ovate, bent like a bow	Bent ventrally
	UW1930Dol	2	96.3 ± 4.8	26.4 ± 2.0	43.4 ± 1.3		
*P. manginii*	UW2082Zgo	30	50.5 ± 1.9	28.4 ± 2.3	11.3 ± 1.5	Flat, wide oval	Bent ventrally
	UW2180Cie	30	45.1 ± 2.0	27.3 ± 2.0	10.0 ± 1.2		
	UW2225IK1	30	48.2 ± 0.0	25.6 ± 0.1	12.8 ± 0.1		
	UW2264INo	25	51.4 ± 2.1	29.1 ± 1.9	11.5 ± 1.1		
*P. minutus*	ACOI 1755	109	26.7 ± 1.2	19.9 ± 1.6	1.9 ± 0.4	Flat, ovoid	Bent dorsally
*P. orbicularis*	UW2362Choc	4	31.8 ± 2.3	23.2 ± 1.0	3.7 ± 0.2	Flat, wide oval	Bent sideways
*P. paraorbicularis*	UW1977Bud2	28	76.6 ± 2.3	44.7 ± 2.3	16.7 ± 2.1	Flat, wide oval	Bent ventrally
	UW1980Oss	3	77.7 ± 0.5	46.3 ± 1.1	14.9 ± 0.6		
*P. pleuronectes*	SAG 1261‐2b	35	51.6 ± 4.8	25.5 ± 2.7	4.7 ± 0.9	Flat, trapezoid‐like	Bent sideways
	UW1981Oss	11	34.5 ± 1.1	23.3 ± 1.1	5.9 ± 0.8		
	UW2007Kro	20	34.2 ± 0.9	21.2 ± 1.0	5.8 ± 0.8		
	UW2202Ban	29	32.4 ± 1.2	23.3 ± 1.2	4.5 ± 0.6		
	UW2211Jel	3	32.9 ± 0.4	23.4 ± 0.1	4.8 ± 0.0		
	UW2230IK1	6	31.2 ± 0.7	22.9 ± 1.1	4.3 ± 0.4		
*P. raciborskii*	ACOI 1758	4**	35.4 ± 1.9	10.5 ± 1.5	8.2 ± 1.5	Flat, cylindrical, spirally twisted	Straight
	UW1778Jez	2	56.8 ± 4.0	13.3 ± 2.1	9.0 ± 0.7		
	UW1815Ur20	8	56.6 ± 1.5	11.0 ± 1.7	11.1 ± 4.6		
	UW2326Gra2	8	57.1 ± 2.3	15.1 ± 1.7	13.4 ± 1.8		
*P. salinus*	UW1914Bud	10	50.9 ± 2.6	35.1 ± 2.6		Egg‐like, not flattened	Lack
	UW1948Wos	3	46.8 ± 4.6	34.0 ± 3.8			
	UW1970Chod	39	47.0 ± 1.6	31.9 ± 2.1			
	UW1983Cie	9	30.8 ± 1.2	21.2 ± 1.4			
	UW2392IK2	107	44.7 ± 3.9	32.7 ± 4.1			
	SAG 1244‐3	109	41.6 ± 1.8	26.7 ± 2.0			
*P. segretii*	ACOI 1337	3	18.4 ± 0.6	12.9 ± 1.4		Thick, wide‐oval	Lack
*P. stokesii*	UW2399Ur19	11	41.3 ± 0.4	30.8 ± 0.4		Thick, wide‐oval	Lack
*P. tenuis*	ACOI 1757	111	32.4 ± 1.5	18.5 ± 1.5	4.4 ± 0.7	Flat, oval	Bent ventrally

*, Based on fig. 1c in Bennett and Triemer 2012; **, based on the photographs from the ACOI website: http://acoi.ci.uc.pt/spec_detail.php?cult_id=1442).

### DNA isolation, amplification, and sequencing

Isolation of total DNA from samples (environmental samples and laboratory cultures of strains) was performed as described previously (Zakryś et al. 2002). An additional step of whole genome amplification was carried out in the case of environmental samples (according to Bennett and Triemer 2012). PCR amplification of the nSSU coding region, purification, and sequencing of the PCR products were performed by standard methods as previously described (Zakryś et al. 2002). To obtain some sequences, the nested PCR method was used as described previously (Łukomska‐Kowalczyk et al. 2020).

### Sequence accession numbers, alignment, and sequence analyses

Fifty‐five new sequences (6 from strains and 49 from environmental isolates) used in the present study were submitted to GenBank with the following accession numbers: MN149548 – MN149602 and MN326770. Information about the accession numbers for the nSSU rDNA sequences reported here and those used for phylogenetic analyses (136 in total, of which 129 for *Phacus*) are shown in Table S1. Identical sequences from different strains/isolates were represented as single terminals in the phylogenetic analyses. The nSSU rDNA sequences were aligned using the MAFFT v.7.2 software (Katoh and Standley 2013) with E‐INS‐I strategy. Alignment was inspected by eye and corrected if necessary. Regions of doubtful homology between sites were removed from the alignments using TrimAl 1.2 with the option “automated1” (Capella‐Gutierrez et al. 2009). After trimming, 2,157 out of 4663 positions were left. Sequence diversity of nSSU rDNA was calculated using the Mega X software (Kumar et al. 2018) as pair‐wise distance based only on unambiguously aligned positions. The alignment and corresponding phylogenetic tree have been submitted to TreeBase (Study Accession URL: http://purl.org/phylo/treebase/phylows/study/TB2:S25743).

#### Phylogenetic analyses

The model of sequence evolution was selected using jModeltest 2.1.7 (Darriba et al. 2012) and the GTR+I+G model was chosen based on AIC, BIC or DT criteria (with ‐lnL = 48697.4; Lanave et al. 1984, Tavare 1986, Rodriguez et al. 1990) and was used to calculate maximum‐likelihood (ML) and Bayesian trees. The ML tree was inferred using RAxML 8.2.11 (Stamatakis 2014) using 1,000 rapid bootstrap inferences.

Bayesian inference (BI) with default priors was performed in MrBayes 3.2.6 software (Ronquist and Huelsenbeck 2003). A gamma correction with eight categories and proportion of invariable sites were used. Two independent runs with four Markov chains were performed. In each run, the chains lasted for 10,000,000 generations and trees were sampled every 1,000 generations. The first 25% of trees were discarded as burn‐in. Convergence among runs was assumed as the average standard deviation of split frequencies was below 0.01. The trees were visualized using FigTree v.1.4.2 (available at http://tree.bio.ed.ac.uk/software).

## RESULTS

### Phylogenetic analyses and morphological characteristics

**Fig. 2:**
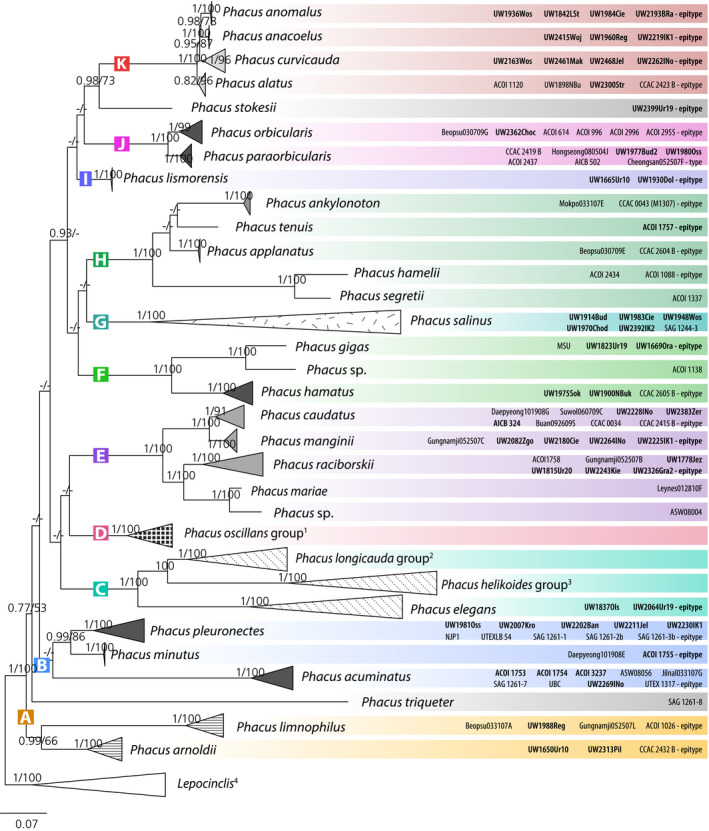
Consensus Maximum Likelihod tree based on 136 nSSU rDNA sequences (of which 129 represent *Phacus*). Isolates or strains with identical nSSU rDNA are represented by single tips. Sequences of the same species are represented by triangles of height proportional to the variability of sequences. Nodes are labeled with the Bayesian posterior probability (pp) values and rapid bootstrap (rbs) values. The pp values <0.75 and rbs values <50 and clades not present in the particular analysis are marked with a hyphen (‐). Scale bar represents number of substitutions per site. Sequences obtained in this study are indicated in bold type. Groups of species: ^1^ *P. brevisulcus* Suwol060709A (type), *P. oscillans* ACOI 1339 (epitype), *P. longisulcus* Psurononuma100609J (type), *P. smulkowskianus* ACOI 1226 (epitype), UW2281Kop, *P. granum* AICB349, *P. polytrophos* CCAC2451B (epitype), *P. minimus* Buan092609I (type), *P. claviformis* Gungnamji052507F (type), *P. hordeiformis* Yongho092609A (type), *P. inflexus* ACOI 1336 (epitype), *P. parvulus* ACOI 1093 (epitype), *P. viridioryza* Sondang060709L (type), *P. pusillus* UTEX 1282 (epitype), *P. skujae* ACOI 1312 (epitype). ^2^ *P. ranula* Jigok090112, *P. convexus* UW1821Ora (epitype), *P. tortus* UW1845Jel (epitype), *P. cordatus* UW1808Ur17 (epitype), *P. longicauda* UW1896Laz (epitype), *P. *sp. Burni081809, *P. circumflexus* UW1844Jel (epitype). ^3^ *P. crassus* UW1566UR15 (type), *P. cristatus* UW1929Dol (type), *P. helikoides* UW1658Ur20 (epitype). ^4^ *L. steinii* Cheongsan052507K5, *L. hispidula* MSU, *L. fusiformis* ACOI 1025, *L. spirogyroides* ACOI 1027, *L. acus* ASW 08037, *L. tripteris* UWOB, *L. fusca* Saeraewool102007P. [Color figure can be viewed at wileyonlinelibrary.com]

Phylogenetic trees obtained by Bayesian (not shown herein) and maximum likelihood methods have a very similar topology, with 11 main clades and two branches with single sequences (Fig. 2, Fig. S1), although the relations between species are not always clearly defined.

The earliest branching clade (labelled as **A**), sister to all other *Phacus* sequences, is not strongly supported, but consists of two maximally supported subclades. It includes sequences of *P. arnoldii* and *P. limnophilus* originated from both laboratory cultures and environmental isolates. The genetic variability of nSSU rDNA does not exceed 4% for either species (Table S2). Representatives of both species morphologically resemble members of genus *Lepocinclis* – the three‐ridged cell shape of *P. arnoldii* makes it similar to *L. tripteris,* while the unflattened cells of *P. limnophilus* are typical for the majority of the representatives of the *Lepocinclis* genus (for details see Discussion). The clade corresponds to clade G (Kim and Shin 2014) and I6 (Kim et al. 2015).

The next clade (**B**), complementing clade F in Kim and Shin (2014) and clade I5 in Kim et al. (2015), includes three strongly supported subclades corresponding to the species: *Phacus pleuronectes*,* P. minutus,* and *P. acuminatus*. The cells of all three species are leaf‐like (flat), widely oval with a short, pointy tail. Sequences of *P. pleuronectes* are located in two groups: one with sequences of only environmental isolates and the second with all six strain sequences (out of which three are identical) and only one environmental sequence, sister to them. All isolates are characterized by a trapezoidal shape of cells in comparison to the ovoid cells of the cultivated strains. The genetic variability in the species does not exceed 3% and is lower than 0.5% among the strain sequences (Table S2). *Phacus acuminatus* and *P. minutus* are morphologically almost undistinguishable (see Taxonomic revisions), but they do not form a common clade and the genetic variability between them varies between 8.1% and 9.6% and is lower than 3% and 0.1% within the species respectively (Table S2).

Strongly supported clade **C** (1/100), that corresponds to clade D1 in Kim and Shin (2014) and to one subclade of the I3 clade in Kim et al. (2015), consists of sequences of ten species characterized by large cells (>75 µm) with long tails. In the clade there are three groups of sequences: (1) representatives of *Phacus ranula, P. convexus, P. tortus, P. longicauda, P. cordatus, P. circumflexus* with one sequence of the strain of unknown morphology (*Phacus* sp. Burni081809), (2) representatives of *P. cristatus, P. crassus, P. helikoides,* and (3) as sister clade to all other sequences, the subclade including two sequences of Polish environmental isolates of *P. elegans* – the species that is represented on the phylogenetic tree for the first time.

Maximally supported (1/100) clade **D** consists of sequences of 14 species closely related to *Phacus oscillans*, which are characterized by a small cell size (<40 µm long) and underwent taxonomic verification earlier (Karnkowska‐Ishikawa et al. 2010, Kim and Shin 2014). The clade corresponds to clade A in Kim and Shin (2014) and to clade I1 in Kim et al. (2015). The new environmental sequence of *P. smulkowskianus* UW2281Kop is located in this clade and it is identical with the sequence of the strain ACOI 1226 (epitype).

Clade **E** (1/100) includes four maximally supported subclades that correspond to four species: *Phacus raciborskii* and *P. mariae* branching together with one sequence of a strain of unknown morphology (*Phacus* sp. ASW 08004) and a pair of sister species: *P. caudatus* and *P. manginii*. Species located in this clade are easily distinguishable on the basis of morphology and their common diagnostic feature is flat cells with a conspicuous dorsal crest, which elongates into a sharp hyaline tail. The *P. manginii* subclade consists exclusively of sequences originated from environmental isolates (from Poland and South Korea), and the other subclades include sequences of both strains and isolates. Genetic diversity in *P. caudatus* and *P. manginii* does not exceed 1.8% and in the *P. raciborskii* clade there are five sequences with their genetic diversity below 0.3% and one (ACOI 1758) that is 4.5–5.0% divergent from all other (Table S2). The E clade corresponds to clade E in Kim and Shin 2014 and to clade I4 in Kim et al. 2015.

Strongly supported clades F, G, and H form a common clade in the maximum likelihood analyses only and in the bayesian analyses, relationships between them are not defined.

Clade **F**, absent in Kim and Shin (2014) and corresponding to a fragment of the I3 clade in Kim et al. (2015), consists of three identical sequences of *Phacus gigas* (one of the strain MSU and two Polish isolates), one sequence of a strain of unknown morphology (*Phacus* sp. ACOI 1138) and a subclade of three *P. hamatus* sequences. The *P. gigas* and *P. hamatus* individuals are flat, wide‐oval in general overview, and terminate with a sharp hyaline tail.

Clade **G**, not present in either Kim and Shin (2014) or in Kim et al. (2015), groups sequences of *Phacus salinus*: five from environmental isolates and one from a cultivated strain. Despite all of them being morphologically indistinguishable (spherical, egg‐like cells with both ends widely rounded), their genetic variability is very diverse (up to 11.3%) and the sequences vary considerably in length. The longest fragment of 18S rDNA belongs to the isolate UW1948Wos (3560 bp), and the sequences of other *P. salinus* isolates and strains are also fairly long: 2,124 bp for strain SAG 1244‐3, 2,151 bp for UW1970Chod, 2,778 bp for UW2392IK2. Usually the length of this fragment varies between 1,900 and 2,050 bp in most other *Phacus* species, and exceeds 2,100 bp only for *P. helikoides, P. crassus, P. cristatus,* and *P. elegans*.

**Fig. 3:**
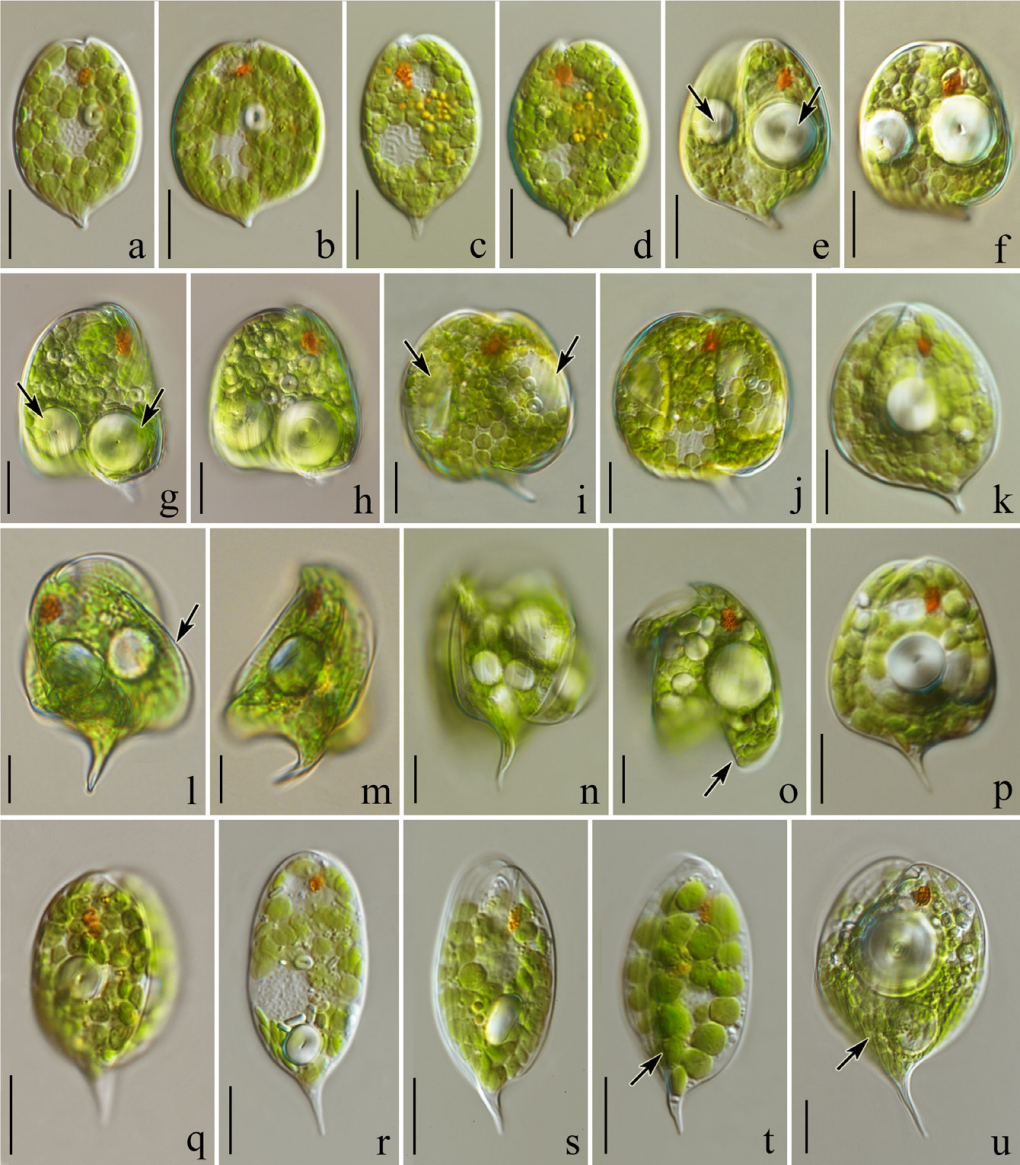
Light microscope photographs showing an overview of living cells of the studied *Phacus* strains (=isolates): (a, b) oval, leaf‐like flat cells of *Phacus acuminatus* (strain UTEX 1317) terminated with a short, sharp, wedge‐shaped tail; (c, d) cells of *P. minutus* (strain ACOI 1755) ending with a tail that is bent toward the dorsal side; (e, f) *P. curvicauda* (isolate UW2262INo), cells with centrally located, large, spherical paramylon grains (arrows); (g, h) trapezoid‐shaped cells of *P. anomalus* (isolate UW1984Cie) with two large, spherical paramylon grains located in the widest and thickest bottom part (arrows); (i, j) two large, parietal, ring‐like paramylon grains visible in the cell of *P. alatus* (strain CCAC 2423 B); (k) *P. orbicularis* (isolate UW2362 Choc); (l–o) spherical cells of *P. anacoelus* (isolate UW2219IK1) with visible crests (arrows) and large, spherical paramylon grains located in the center; (p) trapezoid‐shaped cell of *P. pleuronectes* (isolate UW2211Jel); (q) rotund (triangular when cross‐sectioned) cell of *P. ankylonoton* (strain CCAC 0043) terminated with a straight tail; (r) flat, elongated oval‐like cells of *P. applanatus* (strain CCAC 2604 B) ending with a straight tail; (s, t) cells of*. P. caudatus* (strain CCAC 0034) with a dorsal crest which elongates into a sharp hyaline tail slightly bent toward the ventral side; (u) wide‐oval cell of *P. manginii* (isolate UW2219IK1) with a low crest which elongates into a sharp hyaline tail bent slightly sideways. Scale bars 10 µm.

**Fig. 4:**
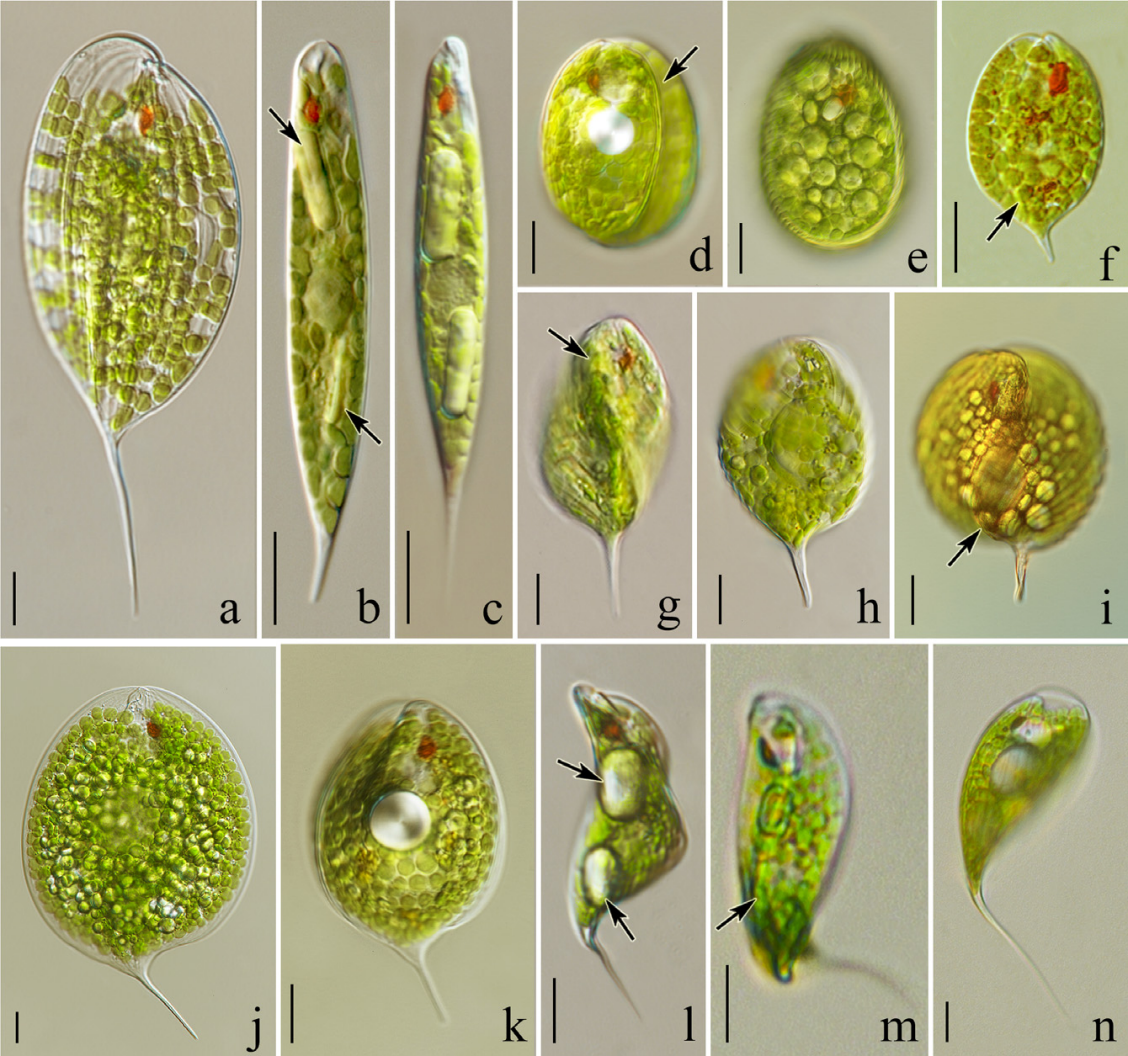
Light microscope photographs showing an overview of living cells of the studied *Phacus* strains (=isolates): (a) elongated oval‐like cell of *Phacus elegans* (isolate UW2064Ur19) elongated with a long, thorn‐like, straight tail; (b, c) spindle‐shaped cells of *P. limnophilus* with two large, rod‐like paramylon grains visible, (b) strain ACOI 1026, (c) isolate UW1988Reg; (d) wide‐oval cell of *P. stokesii* (isolate UW2399Ur19) rounded at both ends; (e) spherical cells of *P. salinus* (strain SAG 1244‐3); (f) *P. tenuis* (strain ACOI 1757), oval cell with a low crest elongated into a tail bent slightly toward the ventral side; (g–i) almost spherical, slightly twisted cells of *P. arnoldii* with three ridges (g, h) strain CCAC 2432B, (i) isolate UW2313Pil; (j) large, flat, almost round cell of *P. gigas* (isolate UW1669Ora) terminated with a sharp tail bent sideways; (k) spoon‐shaped (convex) cell of *P. hamatus* (strain CCAC 2605 B [=ASW 08032]) ending with sharp hyaline tail bent visibly toward the ventral side; (l–m) cylindrical, flat, slightly U‐bent and spirally twisted cells of *P. raciborskii* (isolate UW2326Gra2); (n) cell of *P. lismorensis* (isolate UW1930Dol) bent like a bow, terminated with a long, sharp, pointy tail. Scale bars 10 µm.

Clade **H**, corresponding to the B clade in Kim and Shin (2014) and to clade I2 in Kim et al. (2015), consists of sequences of five species: *Phacus ankylonoton, P. tenuis, P. applanatus, P. hamelii, and P. segretii*, which can be easily distinguished based on morphology and molecular differences (see Figs. 3, q and r, 4f, and also fig. 1, a and b in Kosmala et al. 2007).

Two sequences of environmental isolates of *Phacus lismorensis* form a strongly supported clade **I**, however, that does not define their position. The species has a very characteristic morphology (cells bent like a bow; Fig. 4n) and its sequences were not hitherto present on any phylogenetic tree.

Clade **J** consists of two subclades corresponding to two sister species: *Phacus orbicularis* and *P. paraorbicularis,* which are characterized by wide, ovoid cells ending with a sharp curved tail; both had been previously taxonomically verified (Kosmala et al. 2007, Kim and Shin 2014). Two sequences of Polish isolates of *P. paraorbicularis* and one of *P. orbicularis* are included in the subclades. Variability in the whole clade does not exceed 1.5% (Table S2). The clade corresponds to clade C in Kim and Shin (2014) and to one subclade of the I3 clade in Kim et al. (2015).

Maximally supported clade **K**, corresponding to clade D2 in Kim and Shin (2014) and to one subclade of the I3 clade in Kim et al. (2015), includes sequences of four species. The genetic diversity in that clade is lower than 5% and in most cases (in spite of the variability of the sequence of *Phacus curvicauda* UW2163Wos) does not exceed 0.9% (Table S2). All representatives of the clade are characterized by thick cells with well‐developed ventral and dorsal sides, ending with a short tail curved toward the dorsal side. However, the four species are easily recognizable based on such features as the shape of the large paramylon grains and their location in the cell. Sister clades with the sequences of *P. anacoelus* and *P. anomalus* are maximally supported and the other two subclades have lower support – *P. curvicauda*: 1/96 and *P. alatus*: 0.82/96. All sequences in clade K (aside from the three of *P. alatus*) stem from environmental isolates from Poland.

The single sequence of *Phacus stokesii* has a sister position to clade K, although it is not strongly supported (0.98/73). It represents one out of two (along with *P. segretii*) tailless species (Fig. 4d).

### Taxonomic revisions

Phylogenetic and morphological analyses and a review of the literature enabled the taxonomic identification of almost all clades present on the phylogenetic tree. For new or misidentified clades, as well as those represented by single sequences a taxonomic revision had been conducted. Its basis was to define a species as a group of singular morphotypes, which create a well‐supported clade on the phylogenetic tree. For such clades (morphologically well‐distinguished taxa) diagnostic descriptions were emended, epitypes designated and the nomenclature has been reordered.

Due to the lack of morphological data, the taxonomic affiliation of 5 strains (Jigok090112 as *Phacus ranula* on the phylogeny tree; Leynes012810F as *P. mariae*; Burni081809, ASW 08004 and ACOI1138 as *Phacus* sp.) was not verified.

Given that the isolated cells were destroyed for DNA extraction, the photographs are designated as epitypes (see International Code of Nomenclature for algae, fungi, and plants [Shenzhen Code]; chapter II, section 2, article 9.9; Turland et al. 2018).

**The genus *Phacus*** Dujardin 1841 *. Emend*. Linton and Karnkowska 2010 in Linton et al. 2010, p. 609.

***Phacus acuminatus*** A.Stokes 1885a: 183, fig. 1 (**Fig.** **3a** **and b**)

*Emended diagnosis*: Cells oval, wide‐oval or almost spherical (20–40 × 13–27 µm), leaf–like and flat (with very low crest); cells terminate with a short, sharp, wedge‐shaped, straight or bent sideways tail (on average 2–3 µm long).

*Holotype*: Stokes 1885a, fig. 1

*Epitype:* Figure 3a designated herein that supports the holotype (Stokes 1885a, fig. 1)

*Representative DNA sequence*: GenBank AF286209

*Representative strain:* UTEX 1317

*Type locality:* shallow ponds in Western New York, USA

*Heterotypic synonyms: Phacus acuticauda* Y.V. Roll 1925: 140, 148, pl. 5, fig. 17; *P. acuminatus* subspec. *acuticauda* (Y.V.Roll) Pochmann 1942: 143, fig. 32n; *P. acuminatus* var. *acuticauda* (Y.V.Roll) Huber‐Pestalozzi 1955: 194, fig. 230; *P. acuminatus* subspec. *americana* Pochmann 1942: 141, fig. 32 a‐c; *P. acuminatus* var. *triangulatus* (Y.V.Roll) Svirenko 1938: 56, fig. 60; *P. acuminatus* subspec. *discifera* Pochmann 1942: 143, figs. 32e and f; *P. acuminatus* var. *discifera* (Pochmann) Huber‐Pestalozzi 1955: 193, fig. 225; *P. acuminatus* subspec. *indica* Pochmann 1942: 144, fig. 32p; *P. acuminatus* var. *indica* (Pochmann) Huber‐Pestalozzi 1955: 194, fig. 229; *P. acuminatus* var. *iowensis* Allorge & Jahn 1943: 235, figs. 31, 32; *P. acuminatus* subspec. *javana* Pochmann 1942: 141, fig. 32d; *P. acuminatus* var. *javana* (Pochmann) Huber‐Pestalozzi 1955: 193, fig. 226; *P. acuminatus* subspec. *variabilis* (Lemmermann) Pochmann 1942: 143, figs. 32g, h; *P. brachykentron* Pochmann 1942: 145, fig. 33.

*Comments:* Following the nomenclatural priority rule, this morphological form (cells small, almost spherical, flat, and terminate with a short, sharp, straight or bent sideways, and wedge‐shaped tail) has been assigned the name *Phacus acuminatus*. The individual seen in Stokes' drawing (1885a, fig. 1) corresponds with the aforementioned characteristic, similarly to the cells from the UTEX 1317 strain (chosen as the representative strain), from which the epitype originates. However, due to the high morphological similarity to *P. minutus,* only molecular identification allows the differentiation of the two species. In this situation, designation of epitypes for both species seems justified (see Comments for *P. minutus*). The taxa that constitute *P. acuminatus* synonyms are those whose cells have a morphology similar to *P. acuminatus* and without proper diagnostic traits in their original descriptions.

***Phacus alatus*** G.A.Klebs 1883: 312 (**Fig.** **3** **i and j**)

*Emended diagnosis*: Cells wide‐oval, almost round in general overview (35–46 × 27–34 µm); thick (slightly dorsally flattened); terminate with a short, sharp tail bent dorsally (on average 6–8 µm long). A wide furrow divides the cell in halves, which are slightly twisted longitudinally; two large, ring‐ or shield–like paramylon grains located parietally.

*Basionym*:* Phacus triqueter* in Stein 1878, Der Organismus Infusionsthiere 3, pl. 19 figs. 55‐57

*Lectotype*: designated herein, Stein 1878, pl. 19, fig. 56

*Epitype:* Figure 3i designated herein that supports the lectotype (Stein 1878, pl. 19, fig. 56)

*Representative DNA sequence*: GenBank AJ532474

*Representative strain:* CCAC 2423B (=ASW 08027)

*Type locality:* Germany

*Heterotypic synonyms: Phacus alatus* var. *maximus* Hübner 1886: 6, fig. 8; *P. alatus* var. *lemmermanii* Svirenko 1915a: 117, pl. 3 figs. 6, 7; *P. alatus* var. *latviensis* Skvortsov 1928: 112, pl. 2, fig. 19; *P. lemmermannii* (Svirenko) Skvortsov 1928: 114, pl. 2, figs. 30, 31; *P. alatus* var. *incrassata* Deflandre 1928: 215, figs. 14–17; *P. alatus* var. *maior* Drezepolski 1922: 4; *P. alata* var. *indica* Skvortsov 1937: 73, pl. 9, figs. 17, 18; *P. angulatus* Pochmann 1942: 171, figs. 70a–c; *P. macrostigma* Pochmann 1942: 170, fig. 68; *P. moraviensis* Pochmann 1942: 169, figs. 65, 66; *P. platyaulax* Pochmann 1942: 165, figs. 61–64.

*Comments:* The *P. alatus* individual seen in one of Stein's original drawings (fig. 56) – a thick cell (only slightly flattened, not leaf‐like flat) with a deep furrow and parietal paramylon grains – resembles the Polish representatives of *P. alatus*. Nonetheless, both this cell and others shown in the remaining drawings display a high similarity to *P. curvicauda* and *P. anomalus* due to the presence of a wide furrow dividing the cells, which are slightly twisted longitudinally, in halves. The morphological research presented herein demonstrates that the large paramylon grain location and shape are good diagnostic features for discriminating those three species (for more details see Discussion). The epitype designation for all three species will allow their proper identification. The taxa that constitute synonyms of *P. alatus* are those whose slightly flattened cells posses a furrow and two large, ring‐ or shield–like paramylon grains located parietally.

***Phacus anacoelus*** A.Stokes 1885b: 19, fig. 2 (**Fig.** **3** **l–o**)

*Emended diagnosis*: Cells almost spherical (42–57 × 25–40 µm), slightly flattened, with the ventral and dorsal sides clearly visible; at the back cells terminate with a sharp, hyaline tail (10–12 µm long) bent toward the dorsal side; two high crests run along each side (four crests in sum), and as the cells are spirally twisted, when mobile it seems that the crests are evenly distributed; two large, spherical paramylon grains are located in the center of the cell.

*Holotype:* Stokes 1885b, fig. 2

*Epitype:* Figure 3l designated herein that supports the holotype (Stokes 1885b, fig. 2)

*Representative DNA sequence*: GenBank MN149556

*Type locality:* shallow ponds in Western New York, USA

*Representative locality:* Freshwater, Izdebno Kościelne village, field pond (52°08′21.0″ N, 20°32′03.2″ E)

*Heterotypic synonym: Phacus anacoelus* var. *asiatica* Skvortsov 1958: 164, pl. 3, fig. 27.

*Comments:* The cells of Polish populations are almost spherical with spiral ribs, what is consistent with the original description. However, the individual seen in Stokes's drawing (1885b, fig. 2) also highly resembles the species described later (*P. asymmetricus, P. quinquemarginatus*) (for more details see Discussion). Due to the aforementioned, emending the diagnostic description and designating an epitype will allow its proper identification.

***Phacus ankylonoton*** Pochmann 1942: 148, fig. 37a–e (**Fig.** **3q**).

*Emended diagnosis*: Cells oval (30–50 × 20–30 µm), rotund (triangular when cross‐sectioned), terminate with a straight or slightly ventrally bent, sharp hyaline tail (on average 6‐9 µm long).

*Lectotype*: designated herein, Pochmann 1942, fig. 37d

*Epitype:* Figure 3q designated herein that supports the lectotype (Pochmann 1942, fig. 37d)

*Representative DNA sequence*: GenBank KF744064

*Representative strain:* CCAC 0043

*Type locality:* pond in small town Wolice, Eastern Poland (now Ukraine)

*Comments:* Due to its size and the general cell shape, *P. ankylonoton* is similar to *P. caudatus*, the only difference being the width of the oval cell, which in cross‐section is triangular. This particular trait is represented only in two of Pochmann's drawings (fig. 37d and e), which are in fact an original drawing by Dreżepolski (Dreżepolski 1925, fig. 112 – two cells). It is Dreżepolski's drawing that Pochmann refers to when describing the new taxon – one of them has been designated herein as the lectotype (fig. 37d). The remaining individuals in Pochmann's drawings (37a and b) resemble more *P. caudatus* due to the elongated oval‐like cell shape. The designation of an epitype will allow the proper identification of *P. ankylonoton*.

***Phacus anomalus*** Fritsch & Rich 1929: 73, figs. 24h–n (**Fig.** **3** **g and h**)

*Emended diagnosis*: Cells wide‐egg or trapezoid‐shaped (30–50 × 20–35 µm) in general overview, thick, with a furrow running down the entire length of the cell and dividing it into halves, which are slightly twisted longitudinally; two large, spherical paramylon grains are located in the widest and thickest bottom part of the cell, which elongates into a sharp tail (on average 6–8 µm long) bent toward the dorsal side.

*Lectotype*: designated herein, Fritsch and Rich 1929, fig. 24h

*Epitype:* Figure 3g designated herein that supports the lectotype (Fritsch and Rich 1929, fig. 24h)

*Representative DNA sequence*: GenBank MN149560

*Type locality:* Griqualand West – the interior of South Africa

*Representative locality:* Freshwater, Cielądz village (51°42′50.9" N, 20°20′44.7" E)

*Heterotypic synonyms: Phacus snitkovii* Y.V. Roll 1938: 138, fig. 8; *Phacus anomalus* var. *pullus‐gallinae* Nygaard 1949: 168, fig. 102; *P. drezepolskii* Stawiński 1969: 39 and 50, fig. 78; *P. curvicauda* f. *anomalus* (F.E.Fritsch & M.F.Rich) Safonova in Popova and Safonova 1976: 58, pl. 12, figs. 1–22.

*Comments:* In most of the drawings by Fritsch and Rich (1929) the large, spherical paramylon grains are positioned centrally in the cell, which causes *P. anomalus* to be practically indistinguishable from *P. curvicauda* (both have thick cells with a furrow and the tail bent toward the dorsal side). Meanwhile, the study presented herein proved that in *P. anomalus* the large paramylon grains are located in the widest and thickest, bottom part of the cell. The lectotype designated herein – the cell visible in the drawing 24 h (Fritsch and Rich 1929) has the most antapical paramylon grains (located the closest to the posterior of the cell). Taxa that constitute *P. anomalus* synonyms are those whose cell morphology corresponds to the emended diagnostic description (for more details see Discussion).

***Phacus applanatus*** Pochmann 1942: 152, fig. 42 (**Fig.** **3r**)

*Emended diagnosis*: Cells elongated oval–like (40 × 20 µm on average), flat, terminate with a rather long, straight tail (6–7 µm on average).

*Holotype*: Pochmann 1942, fig. 42

*Epitype:* Figure 3r designated herein that supports the holotype (Pochmann 1942, fig. 42)

*Representative DNA sequence*: GenBank EU624031

*Representative strain:* CCAC 2604 B

*Type locality:* Sandberg pond near Teplitz‐Schönau, Germany

*Comments:* The morphological study of the CCAC 2604B strain (isolated in the Netherlands, channel in Leiden) shows that its representatives have cells terminated with a tail that is twice as long (6–7 µm) in comparison with the individual in the drawing by Pochmann (1942, fig. 42).

***Phacus arnoldii*** Svirenko 1915a: 120 and 131, pl. 3, fig. 1 (**Fig.** **4** **g‐i**)

*Emended diagnosis*: The shape of the organism is particularly peculiar, due to the three wings (ridges) of the almost spherical and slightly twisted cells (46–95 × 25–69 µm); cells end with a hyaline tail (12–23 µm); in cross section the cells appear triangular with concave sides (similarly to *L. tripteris*); periplast spirally striated with numerous, perpendicular struts between longitudinal periplast strips; the longitudinal periplast strips are wide and sparsely arranged.

*Holotype:* Svirenko 1915a, pl. 3, fig. 1

*Epitype:* Figure 4g designated herein that supports the holotype (Svirenko 1915a, pl. 3, fig. 1).

*Representative DNA sequence*: GenBank GQ422793

*Representative strain:* CCAC 2432B (=ASW 08064)

*Type locality:* Kharkiv district, Zmiewsk, Krugloje Lake, Russia (currently Ukraine)

*Heterotypic synonyms: Phacus warszewiczii* Drezepolski 1925: 235, fig. 125; *P. arnoldii* var. *ovatus* T.G.Popova 1947: 57, pl. 2, fig. 12.

*Comments:* In the original description, “round, flat cells with a high, S‐shaped crest” and “wide, sparsely arranged periplast strips” are mentioned – thanks to these features the Polish strains could be identified. As our research has shown, cells appear triangular with concave sides in cross section that gives the impression of three wings (ridges) and is a diagnostic trait of *P. arnoldii*. Meantime, in Svirenko's drawing (1915a, pl. 3, fig. 1) only a high crest is visible that does not looks like one of the three equally sized wings. Furthermore, we have shown that a more or less round shape of the cells has no diagnostic meaning, which is why *P. arnoldii* var. *ovatus* has been included as a synonym of *P. arnoldii*.

***Phacus brevisulcus*** Kim & Shin 2014: 955, fig. 2B

*Type:* (see Kim and Shin 2014), permanently preserved material from the strain Suwol060709A deposited at the Chungnam National University, as number CNU 025427. Figure 2B in Kim and Shin 2014 shows the type.

*Representative DNA sequence:* GenBank KF744067

***Phacus caudatus*** Hübner 1886: 5, fig. 5 (**Fig.** **3** **s and t**)

*Emended diagnosis*: Cells elongated oval–like (31–47 × 15–30 µm), slightly asymmetrical, flat, with a high dorsal crest which elongates into a sharp hyaline tail (on average 4–8 µm), slightly bent toward the ventral side; periplast longitudinally striated.

*Holotype*: Hübner 1886, fig. 5

*Epitype:* Figure 3t designated herein that supports the holotype (Hübner 1886, fig. 5)

*Representative DNA sequence*: GenBank AJ532482

*Representative strain:* CCAC 2415B (=ASW 08020)

*Type locality:* Germany, Stralsund

*Heterotypic synonyms: Phacus caudatus* var. *undulata* Skvortsov 1922: 191, fig. 7; *P. caudatus* var. *lata* Allorge and Lefèvre 1925: 126, figs. 18–21; *P. caudatus* var. *minor* Drezepolski 1925: 230, fig. 107; *P. caudatus* var. *ovalis* Drezepolski 1925: 231, fig. 111; *P. ovalis* Skvortsov 1958: 165, pl. 3, fig. 33; *P. caudatus* var. *volicensis* Drezepolski 1925: 230, fig. 105.

*Comments: Phacus caudatus* is very similar to *P. manginii* and *P. ankylonoton* due to the presence of a crest that elongates into the hyaline tail. Meanwhile, both the diagnostic description as well as Hübner's drawing of *P. caudatus* (Hübner 1886, fig. 5) does not contain any diagnostic features that would allow the distinguishment of *P. caudatus* from the two species described below (for more details see Discussion). Due to the aforementioned, the designation of an epitype seems justified. The taxa that constitute *P. caudatus* synonyms are those whose cell morphology corresponds to the emended diagnosis.

***Phacus circumflexus*** Pochmann 1942: 206, fig. 119a–f

*Epitype:* Łukomska‐Kowalczyk et al. 2015, fig. 3k

*Representative DNA sequence:* GenBank KP944083

***Phacus claviformis*** Kim & Shin 2014: 955, fig. 2E

*Type:* (see Kim and Shin 2014), permanently preserved material from the strain Gungnamji052507F deposited at the Chungnam National University, as number CNU 025429. Figure 2E in Kim and Shin 2014 shows the type

*Representative DNA sequence:* GenBank KF744071

***Phacus convexus*** (Pochmann) Zakryś & M.Łukomska, **nom. nov.**

*Nomen novum*:* Phacus convexus* [≡*Phacus longicauda* (Ehrenb.) subs *P. rotundus* Pochmann, Pochmann 1942, Arch. Protistenk. 95: 201, figs. 111a–e], non *Phacus rotundus* Brabez 1941, Beihefte zum Botanischen Centralblatt 61: 220, fig. 13b.

*Lectotype:* Stein 1878, pl. 20, fig. 2 (Łukomska‐Kowalczyk et al. 2015)

*Epitype:* Łukomska‐Kowalczyk et al. 2015, fig. 3g

*Representative DNA sequence:* GenBank KP944090 (Łukomska‐Kowalczyk et al. 2015)

*Representative locality*: Freshwater, pond in Oracze village (53°52′36.6" N, 22°20′41.2" E; Łukomska‐Kowalczyk et al. 2015)

*Comments:* The nomenclatural combination *P. rotundus* (Pochmann) Zakryś & M.Łukomska proposed by Łukomska‐Kowalczyk et al. (2015) for *P. longicauda* (Ehrenberg) subs. *rotundus* Pochmann (1942) is invalid, as a previously described taxon of the same name (though of a different morphology from *P. longicauda* subs. *rotundus*) exists (*P. rotundus* Brabez 1941). We propose *P. convexus nomen novum* for the morphological form known previously as *P. longicauda* subs. *rotundus*. The latin name *convexus* refers to the spoon‐shaped (convex) cells.

The proposed lectotype, Stein's drawing (1878 (pl. 20, fig. 2), was chosen not only because it is one of the few Pochmann (1942, *p. *201) referred to when describing the subspecies *rotundus*, but also mainly because Stein (1878) was the first who noticed and documented the occurrence of various morphological forms of *P. longicauda* (pl. 20, figs. 1–3 in Stein 1878), including the form “rotunda” (pl. 20, fig. 2 in Stein 1878)” (Łukomska‐Kowalczyk et al. 2015). However, as we have no drawing, nor was the convex cell shape ever mentioned in Stein's diagnostic description for the “rotunda” form, we believe an epitype designation is justified.

***Phacus cordatus*** (Pochmann) Zakryś & M.Łukomska 2015: 1151, fig. 3a–c.

*Epitype:* Łukomska‐Kowalczyk et al. 2015, fig. 3b

*Representative DNA sequence:* GenBank KP944102

***Phacus crassus*** Zakryś & M.Łukomska 2015: 1153, fig. 3o and p

*Type:* Łukomska‐Kowalczyk et al. 2015, fig. 3p

*Representative DNA sequence:* GenBank KP944108

***Phacus cristatus*** Zakryś & M.Łukomska 2015: 1153, fig. 3q

*Type:* Łukomska‐Kowalczyk et al. 2015, fig. 3q

*Representative DNA sequence:* GenBank KP944109

***Phacus curvicauda*** Svirenko 1915a: 117 and 130, pl. 3 figs. 13–16 (**Fig.** **3** **e and f**)

*Emended diagnosis*: Cells wide‐oval or wide egg‐shaped (22–37 × 16–27 µm), thick, divided into two longitudinally twisted parts by a furrow; terminate with a sharp, short hyaline tail bent toward the dorsal side (on average 2.5–5 µm); two large, spherical paramylon grains positioned centrally in the cell.

*Lectotype:* designated herein, Svirenko 1915a, fig. 13.

*Epitype:* Figure 3e designated herein that supports the lectotype (Svirenko 1915a, fig. 13)

*Representative DNA sequence*: GenBank MN149567

*Type locality:* wetlands and small lakes near Kharkov, Russia (now Ukraine)

*Representative locality:* Freshwater, Izdebno Nowe village, pond (52°08′01.3″ N 20°32′52.9″ E)

*Heterotypic synonym: Phacus curvicauda* f. *robusta* Allorge & Lefèvre 1925: 127 figs. 50 and 51.

*Comments:* In Svirenko's drawings, the large paramylon grains are located centrally in the cells, which allowed us to identify the Polish strains of *Phacus curvicauda*. However, in cell shape, *P. curvicauda* is very similar to *P. anomalus* and *P. alatus* (they all have thick cells with a long furrow). The best diagnostic trait for differentiating the three species is cell shape, as well as the location and shape of the large paramylon grains (for more details see Discussion and Comments for *P. anomalus* and *P. alatus*). The drawing indicated as the lectotype (Svirenko 1915a, fig. 13) shows a long furrow and round paramylon grains – in the remaining images (figs. 14, 15, 16), the individuals either have short furrows, or ring‐like paramylon grains. The designation of an epitype will allow the proper identification of *P. curvicauda*.

***Phacus elegans*** Pochmann 1942: 199, fig. 107 (**Fig.** **4a**)

*Emended diagnosis*: Cells elongated oval‐like (112–147 × 38–51 µm), with a low crest which elongates into a long, thorn‐like, straight hyaline tail (on average 40–50 µm); the front of the cell visibly asymmetrical; cell straightened out both when immobile and swimming.

*Holotype*: Pochmann 1942, fig. 107.

*Epitype:* Figure 4a designated herein that supports the holotype (Pochmann 1942, fig. 107)

*Representative DNA sequence*: GenBank MN149571

*Type locality:* Southern Germany, peatbog Rauhen Wiese near Böhmenkirch (Schwarzwald)

*Representative locality:* Freshwater, Urwitałt, pond 19 (53°50′38.0" N, 21°38′06.5" E)

*Comments:* The Polish populations of this species have been identified based on the shape of the cells (elongated oval‐like with a visibly asymmetrical anterior end, ending with a long hyaline tail). According to Pochmann, *Phacus elegans* can be distinguished from *P. lismorensis* based on “a more elongated cell form and a shorter tail.” Furthermore, we have established that the presence of a crest that elongates into a long, straight hyaline tail is a diagnostic morphological feature; in *P. lismorensis* representatives the tail is ventrally bent (for more details see Discussion).

***Phacus gigas*** A.M.Cunha 1913: 110, pl.10, fig. 3 (**Fig.** **4j**).

*Emended diagnosis*: Cells almost round (90–132 × 46–84 µm), flat, posterior visibly asymmetrical; cells terminate with a sharp hyaline tail (on average 20–28 µm long), which is bent sideways. Periplast longitudinally striated.

*Holotype:* Cunha 1913, pl.10, fig. 3

*Epitype:* Figure 4j designated herein that supports the holotype (Cunha 1913, pl.10, fig. 3)

*Representative DNA sequence*: GenBank MN149572

*Type locality:* Manguinhos, Rio de Janeiro

*Representative locality:* Freshwater, Oracze, farm pond (53°52′36.6" N, 22°20′41.2" E)

*Comments: Phacus gigas* has large, flat, almost round cells, and the Polish populations have been identified based on these traits. The study presented herein shows that the diagnostic feature allowing the proper identification of *P. gigas* (and therefore making the differentiation between other similar species, e.g., *P. hamatus, P. paraorbicularis,* or *P. orbicularis* possible) is the posterior end asymmetry as well as the tail bent sideways. As the cell asymmetry is not mentioned either in the diagnostic description or in Cunha's drawing (1913, pl.10, fig. 3), we believe that the designation of an epitype is justified.

***Phacus granum*** Drezepolski 1925: 231 and 266, pl. 3, fig. 119.

Cells small (18–23 × 7.5–12 µm), cylindrical, slightly flattened, narrowing at both ends, terminate with a blunt papilla (see Karnkowska‐Ishikawa et al., 2010).

*Representative DNA sequence*: GenBank DQ249880

*Representative strain:* AICB 349

***Phacus hamatus*** Pochmann 1942: 182, fig. 86 a–f (**Fig.** **4k**)

*Emended diagnosis*: Wide egg–like, spoon‐shaped (convex) cells (35–66 × 25–43 µm) ending with a sharp hyaline tail (on average 7.5–15 µm long). The tail is visibly bent toward the ventral side, which causes the cells not to adhere to the substrate, but rather “lean” on the tail. Periplast longitudinally striated.

*Lectotype*: designated herein, Pochmann 1942, fig. 86b.

*Epitype:* Figure 4k designated herein that supports the lectotype (Pochmann 1942, fig. 86b)

*Representative DNA sequence*: GenBank AJ532473

*Representative strain:* CCAC 2605B (=ASW 08032)

*Type locality:* pond in small town Dobrostany, Eastern Poland (now Ukraine)

*Comments:* The characteristic shape of the *Phacus hamatus* cells (spoon‐shaped [convex]) visible in all of Pochmann's drawings (1942, fig. 86, a–f) allowed the identification of the species. On the other hand, the cell asymmetry visible in all drawings and stressed in the original diagnosis, is confusing, as our research has shown that it concerns only *P. gigas*. *Phacus hamatus* has symmetrical cells, but both species have so far been difficult to distinguish and often are confused (for more details see Discussion). The individual indicated here as the lectotype (fig. 86b in Pochmann 1942) has the least visible cell asymmetry.

***Phacus hamelii*** Allorge & Lefèvre 1925: 128, figs. 55–57.

*Epitype:* designated herein, Kosmala et al. 2007, fig. 1a.

*Representative DNA sequence:* GenBank DQ397673 *Comment:* As the epitypification statement published in Kosmala et al. 2007 (p. 1078)) does not include the phase “designated here” (or on equivalent), thus the nomenclatural act has not been effected in accordance with ICN Art. 7.11 (Turland et al. 2018) and is conducted herein.

***Phacus helikoides*** Pochmann 1942: 212, figs. 124, 125.

*Epitype:* Łukomska‐Kowalczyk et al. 2015, fig. 3r

*Representative DNA sequence:* GenBank KP944094

***Phacus hordeiformis*** Kim & Shin 2014: 956, fig. 2, F and G

*Type:* (see Kim and Shin 2014), permanently preserved material from the strain Yongho092609A deposited at the Chungnam National University, as number CNU 025430. figures 2F and 2D in Kim and Shin 2014 show the type.

*Representative DNA sequence:* GenBank KF744073

***Phacus inflexus*** (Kisselev) Pochmann 1942: 133, fig. 20 a–h

*Epitype:* designated herein, Karnkowska‐Ishikawa et al. 2010, fig. 1b.

*Representative DNA sequence:* GenBank FJ590503 *Comment:* As the epitypification statement published in Karnkowska‐Ishikawa et al. 2010 (p. 178) does not include the phrase “designated here” (or an equivalent), thus the nomenclatural act has not been effected in accordance with ICN Art. 7.11 (Turland et al. 2018) and is conducted herein.

***Phacus limnophilus*** (Lemmermann) E.W.Linton & Karnkowska in Linton et al. 2010: 609 (**Fig.** **4** **b and c**)

*Emended diagnosis*: Cells spindle‐shaped or cylindrical (45–90 × 7–13 µm), not flattened (round in cross section); posterior end narrowed and sharply ended; two large, rod–like paramylon grains – one placed in front of the nucleus, the other behind it.

*Basionym: Euglena limnophila* Lemmermann, 1898, *Botanisches Centralblatt* 76: 152

*Holotype:* Lemmermann 1913 in Pascher's Süssw.‐Fl., fig. 205.

*Epitype:* Figure 4b designated herein that supports the holotype (Lemmermann 1913 in Pascher's Süssw.‐Fl., fig. 205)

*Representative DNA sequence*: GenBank DQ249877

*Representative strain:* ACOI 1026

*Type locality:* Germany, ponds in Düsseldorf, Grimma, Gohlis and Knautheim near Leipzig

*Comments:* Both the diagnosis and drawing (Lemmermann 1898, 1913, fig. 205) are confusing in terms of the number and location of the large paramylon grains – the description mentions one grain positioned at the back of the cell (behind the nucleus) or two grains placed at the nucleus's level on both sides (the left and the right). The second option is depicted in Lemmermann's drawing (1913, fig. 205). Meanwhile the rod‐like, large paramylon grains always exist as a pair – one in front of the nucleus, and the other behind; there is no room in the narrow, cylindrical cells of *Phacus limnophilus* for the paramylon grains to be located on nucleus level. Many euglenid researchers, including ourselves, have noted this (Pringsheim 1956, Popova and Safonova 1976, Tell and Conforti 1986 among others).

***Phacus lismorensis*** Playfair 1921: 125, pl. 5 fig. 14 (**Fig.** **4n**)

*Emended diagnosis*: Cells long ovate (70–134 × 24–40 µm), flat (no crest), bent like a bow; anterior end wide and rounded; posterior end gradually narrowed and terminated with a long, sharp, pointy tail (on average 40–50 µm long); the tail is set almost at a right angle and does not straighten even when the cells are immobile (not swimming).

*Holotype:* Playfair 1921, pl. 5 fig. 14.

*Epitype:* Figure 4n designated herein that supports the holotype (Playfair 1921, pl. 5 fig. 14)

*Representative DNA sequence*: GenBank MN149578

*Type locality:* Australia, neighbourhood of Lismore, freshwater pond

*Representative locality:* Freshwater, Doliwy, fish pond (54°01′55.5″ N, 22°18′02.0″ E)

*Heterotypic synonyms: Phacus rostafińskii* Drezepolski 1922: 13, fig. 3, a and u. *Phacus pediformis* Skvortsov 1958: 163, pl. 3, fig. 30.

*Comments:* Polish reresentatives of *Phacus lismorensis* have a long tail heavily bent toward the ventral side. In Playfair's drawing, it is only slightly bent which is why *P. lismorensis* might be mistaken with *P. elegans,* to which it is very similar both in cell shape and size (for more details see Discussion and *Comments* for *P. elegans*). The taxa that constitute *P. lismorensis* synonyms are those whose cells are long ovate, flat and terminate with a long tail heavily bent ventrally.

***Phacus longicauda*** (Ehrenberg) Dujardin 1841: 337, pl. 5 fig. 6.

*Epitype:* Łukomska‐Kowalczyk et al. 2015, fig. 3d

*Representative DNA sequence:* GenBank KP944103

***Phacus longisulcus*** Kim & Shin 2014: 955, fig. 2A

*Type:* (see Kim and Shin 2014), permanently preserved material from the strain Psurononuma100609J deposited at the Chungnam National University, as number CNU 025426. Figure 2A in Kim and Shin 2014 shows the type.

*Representative DNA sequence:* GenBank KF744079

***Phacus manginii*** Léfévre 1931: 352, pl. 2, figs. 39–46 (**Fig.** **3u**)

*Emended diagnosis*: Cells wide oval‐ or egg‐like (38–55 × 22–33 µm) with a well‐developed dorsal fold (crest) that elongates into a sharp hyaline tail (on average 10–13 µm long) slightly bent ventrally; periplast longitudinally striated.

*Lectotype*: designated herein, Léfévre 1931, fig. 40.

*Epitype:* Figure 3u designated herein that supports the lectotype (Léfévre 1931, fig. 40)

*Representative DNA sequence*: GenBank MN149581

*Representative locality:* Freshwater, Izdebno Kościelne village, field pond (52°08′21.0″ N, 20°32′03.2″ E)

*Type locality:* Indochina

*Comments:* Polish populations of *Phacus manginii* have been identified based on cell size as well as the presence of a well‐developed dorsal fold elongated into a sharp tail. The well‐developed dorsal fold makes *P. manginii* similar to *P. caudatus*. Moreover the differentiation of the two species is further complicated by the fact, that in two of Lefèvre's drawings (figs. 39 and 43) the cells of *P. manginii* are asymmetrical, while it is the representatives of *P. caudatus* that possess asymmetrical cells (for more details see Discussion). In such a situation, indicating a lectotype (fig. 40 in Léfévre 1931 displaying a symmetrical *P. manginii* cell) and designating an epitype will allow the proper identification of this species.

***Phacus minimus*** Kim & Shin 2014: 955, fig. 2D

*Type:* (see Kim and Shin 2014), permanently preserved material from the strain Buan092609I deposited at the Chungnam National University, as number CNU 025428. figure 2D in Kim and Shin 2014 shows the type.

*Representative DNA sequence:* GenBank KF744081

***Phacus minutus*** (Playfair) Pochmann 1942: 182, fig. 85 (**Fig.** **3** **c and d**)

*Emended diagnosis*: Cells flat, ovoid (20–30 × 11–28 µm), ending with an inconspicuous tail (on average 1‐2 long µm) that is bent toward the dorsal side. Periplast longitudinally striated.

*Holotype:* Pochmann 1942, fig. 85.

*Epitype:* Figure 3d designated herein that supports the holotype (Pochmann 1942, fig. 85)

*Representative DNA sequence*: GenBank MN149551

*Representative strain:* ACOI 1755

*Type locality:* Australia, Sydney, botanical garden

*Comments:* Strain ACOI 1755 has been identified as *Phacus minutus* due to its shape and cell size. Meanwhile, the morphological study presented has shown, that *P. minutus* has a tail bent toward the dorsal side that makes it different from *P. acuminatus* (bent sideways). Both species are very similar in size and shape, which is why the designation of their epitypes seems justified (for more details see Discussion).

***Phacus orbicularis*** Hübner 1886: 5, fig. 1 (**Fig.** **3k** **)**

*Epitype:* herein designated, Kosmala et al. 2007, fig. 1h.

*Representative DNA sequence:* GenBank DQ397671

*Heterotypic synonym: Phacus heimii* M.Lefèvre 1933: 261, figs. 11‐13.

*Comments: Phacus heimii* has been included as a synonym of *P. orbicularis*, as the sequence of the Polish isolate (UW2362Choc), the cells of which matched the morphological form described as *P. heimii*, was located in the *orbicularis* clade (for more details see Discussion). As the epitypification statement published in Kosmala et al. 2007 (p. 1077) does not include the phrase “designated here” (or an equivalent), thus the nomenclatural act has not been effected in accordance with ICN Art. 7.11 (Turland et al. 2018) and is conducted herein.

***Phacus oscillans*** G.A.Klebs 1883: 313, pl. 3, fig. 6

*Epitype:* designated herein, Karnkowska‐Ishikawa et al. 2010, fig. 1h.

*Representative DNA sequence:* GenBank FJ590499 *Comment:* Because the epitypification statement published in Karnkowska‐Ishikawa et al. 2010 (p. 177) does not include the phrase “designated here” (or an equivalent), thus the nomenclatural act had been not effected in accordance with ICN Art. 7.11 (Turland *et al*. 2018) and that's why it's done now.

***Phacus paraorbicularis*** Kim & Shin 2014: 956, fig. 2J

*Type:* (see Kim and Shin 2014), permanently preserved material from the strain Cheongsan052507F deposited at the Chungnam National University, as number CNU 025432. Figure 2J in Kim and Shin 2014 shows the type.

*Representative DNA sequence:* GenBank KF744083

***Phacus parvulus*** G.A.Klebs 1883: 313, pl. 3, fig. 5

*Epitype:* designated herein, Karnkowska‐Ishikawa et al. 2010, fig. 1p.

*Representative DNA sequence:* GenBank AJ532472 *Comment:* Because the epitypification statement published in Karnkowska‐Ishikawa et al. 2010 (p. 177) does not include the phrase “designated here” (or an equivalent), thus the nomenclatural act had been not effected in accordance with ICN Art. 7.11 (Turland *et al*. 2018) and that's why it's done now.

***Phacus pleuronectes*** (O.F.Müller) Nitzsch ex Dujardin 1841: 336, pl. 5, fig. 5a and b, 1841 (**Fig.** **3p**)

*Emended diagnosis*: Cells ovoid or trapezoid‐like (30–55 × 20–28 µm).

*Epitype:* designted herein, Kosmala et al. 2007, fig. 1d.

*Representative DNA sequence:* GenBank AJ532475

*Heterotypic synonyms: Phacus trapezoides* Stawiński 1969: 39 and 50, fig. 76 a and b; *P. trapezialis* Shi et al., 1999: 264; pl. 69, figs. 3–5.

*Comments:* The names *Phacus trapezoides* and *P. trapezialis* have been included as synonyms of *P. pleuronectes*, as all five sequences of the Polish cells had a trapezoid–like shape and are in the *pleuronectes* clade (for more details see Discussion).

***Phacus polytrophos*** Pochmann 1942: 128, fig. 15 a–d

*Epitype:* designated herein, Karnkowska‐Ishikawa et al. 2010, fig. 1m.

*Representative DNA sequence:* GenBank FJ590498 *Comments:* As the epitypification statement published in Karnkowska‐Ishikawa et al. 2010 (p. 178) does not include the phrase “designated here” (or an equivalent), thus the nomenclatural act has not been effected in accordance with ICN Art. 7.11 (Turland et al. 2018) and is conducted herein.

***Phacus pusillus*** Lemmermann 1910: 514

*Epitype:* designated herein, Karnkowska‐Ishikawa et al. 2010, fig. 1r.

*Representative DNA sequence:* GenBank AF190815 Comment: As the epitypification statement published in Karnkowska‐Ishikawa et al. 2010 (p. 178) does not include the phrase “designated here” (or an equivalent), thus the nomenclatural act has not been effected in accordance with ICN Art. 7.11 (Turland et al. 2018) and is conducted herein.

***Phacus raciborskii*** Drezepolski 1925: 234 and 266, fig. 113 (**Fig.** **4** **l and m**)

*Emended diagnosis*: Cells flat, cylindrical (35–60 × 8–15 µm), slightly U‐bent and spirally twisted; anterior rounded, narrowing toward the end in a wedge‐like manner and terminated with a sharp hyaline tail (on average 9–13 µm long); low crest running along the entire cell length; periplast longitudinally striated. Two large, ring‐like paramylon grains with one placed in front of the nucleus, and the other behind it.

*Holotype*: Dreżepolski 1925, fig. 113.

*Epitype:* Figure 4m designated herein that supports the holotype (Dreżepolski 1925, fig. 113)

*Representative DNA sequence*: GenBank MN149594

*Type locality:* pond in small town Dobrostany, Eastern Poland (now Ukraine)

*Representative locality:* Freshwater, Grabówek village, pond (53°50′52.9" N, 21°42′19.6" E)

*Heterotypic synonyms*:* Phacus raciborskii* var. *triqueter* Z.X.Shi 1987: 358, fig. 1n–p; *P. trimarginatus* Allorge & Jahn 1943: 242, figs. 33–35.

*Comments:* Following the priority rule, the representatives of Polish populations that displayed the characteristic cell shape (cylindrical, flat, with a low crest along the entire cell length, U‐bent and spirally twisted) are known as *Phacus raciborskii,* while the names of taxa with a morphology identical to *P. raciborskii*, but described later, have been included as its synonyms (for more details see Discussion).

***Phacus salinus*** (F.E.Fritsch) E.W.Linton & Karnkowska in Linton et al. 2010: 609 (**Fig.** **4e**)

*Emended diagnosis*: Cells wide egg‐like (32–57 × 25–48 µm), spherical (not flattened), widely rounded at the posterior end; lateral reservoir opening; wide periplast striation running spirally from right to left. Paramylon grains monomorphic (only small ones), oval and ring‐like.

*Representative DNA sequence*: GenBank EU624028

*Representative strain:* SAG 1244‐3

*Comments:* A new diagnostic trait – the lateral reservoir opening – has been included in the description. Moreover the measurements of the cells of the Polish populations have been taken into account, together with literature data. This seems justified, particularly that no such emendment had been done by Linton et al. (2010) when moving *Phacus salinus* from *Lepocinclis* to *Phacus*.

***Phacus segretii*** Allorge & Lefèvre 1925: 128, figs. 58–60.

Cells wide‐oval (22–28 × 20–22 µm), thick, narrowed, and rounded on both sides, without a tail; the apical furrow almost reaches the posterior end; large, flat paramylon grain located in the center of the cell; periplast longitudinally striated (Pochmann 1942; ).

*Representative DNA sequence*: GenBank FJ719635

*Representative strain:* ACOI 1337

*Heterotypic synomym*:* Phacus stokesii* f. *minor* W.Conrad 1938: 12, figs. 45–47.

*Comments:* The small cells of *Phacus stokesii* f. *minor* indicate that it is a representative of *P. segretii*, which is why the name *P. stokesii* f. *minor* has been included as a synonym of *P. segretii*. The main difference between the two species is the size (*P. stokesii:* 40–46 × 30–35 µm; *P. segretii*: 22–28 × 20–22 µm).

***Phacus skujae*** Skvortsov 1928: 116, pl. 2, fig. 42.

*Epitype:* designated herein, Karnkowska‐Ishikawa et al. 2010, fig. 1j.

*Representative DNA sequence:*FJ597146 *Comment:* As the epitypification statement published in Karnkowska‐Ishikawa et al. 2010 (p. 178) does not include the phrase “designated here” (or an equivalent), thus the nomenclatural act has not been effected in accordance with ICN Art. 7.11 (Turland et al. 2018) and is conducted herein.

***Phacus smulkowskianus*** (Zakryś) W.H.Kusber 1998: 246.

*Epitype:* designated herein, Karnkowska‐Ishikawa et al. 2010, fig. 1e.

*Representative DNA sequence:* GenBank DQ249881 *Comment:* As the epitypification statement published in Karnkowska‐Ishikawa et al. 2010 (p. 179) does not include the phrase “designated here” (or an equivalent), thus the nomenclatural act has not been effected in accordance with ICN Art. 7.11 (Turland et al. 2018) and is conducted herein.

***Phacus stokesii*** Lemmermann 1901:88, 1910: 518, fig. 9 **(Fig.** **4d** **)**

*Emended diagnosis*: Cells wide‐oval (40–46 × 30–35 µm), thick, widely rounded at both ends, without a tail; apical furrow deep, running along the entire cell length; large, plate**–**like paramylon grain located in the center of the cell; periplast longitudinally striated.

*Holotype:* Lemmermann 1910, fig. 9

*Epitype:* Figure 4d designated herein that supports the lectotype (Lemmermann 1910, fig. 9)

*Representative DNA sequence*: GenBank MN149602

*Type locality:* North America, Teichen, ponds

*Representative locality:* Freshwater, Urwitałt village, field pond 19 (53°50'38.0" N, 21°38′06.5" E)

*Heterotypic synonyms: Phacus aspidion* Pochmann 1942: 193, fig. 99; *P. balatonicus* Hortobágyi 1943: 87, pl. 2, figs. 27–36; *P. fominii* Y.V. Roll 1938: 137, fig. 1; *P. starmachii* Stawiński 1969: 37 and 50, fig. 77; *P. betkowski* Stawiński 1969: 39 and 49, fig. 67a and b.

*Comments:* The morphology of the cells of Polish populations of *Phacus stokesii* (thick, widely rounded at both ends, without a tail) corresponds to the original diagnosis. The drawing by Lemmermann (1913, fig. 231), on the other hand, is confusing as it shows an almost round form, while in reality the shape of the cells of this species is wide‐oval. The taxa that constitute *P. stokesii* synonyms have wide‐oval cells (40–46 × 30–35 µm), thick and rounded at both ends (for more details see Discussion).

***Phacus tenuis*** Svirenko 1915b: 336, pl. 2 figs. 17 and 18 (**Fig.** **4f**)

*Emended diagnosis*: Cells oval (29–43 × 15–25 µm), flat, with a low crest that elongates into a sharp hyaline tail (3–6 µm), which is straight or slightly bent toward the ventral side.

*Lectotype:* designated herein, Svirenko 1915b, fig. 17.

*Epitype:* Figure 4f designated herein that supports the lectotype (Svirenko 1915b, fig. 17)

*Representative DNA sequence*: GenBank MN149552

*Representative strain:* ACOI 1757

*Type locality:* Republic of Armenia, Bogdan Lake and pond located close to city Echmiadzin

*Homotypic synonym: Phacus caudata* var. *tenuis* Svirenko 1915a: 121, pl. 3, figs. 17 and 18;

*Heterotypic synonym*:* Phacus caudatus* var. *minor* Drezepolski 1925: 230, fig. 107.

*Comments:* According to Svirenko (1915b) *P. tenuis* differs from *P. caudatus* by having more oval cells and a lower crest (the latin name “tenuis” means flat), and this has been confirmed by our research. Meanwhile the individuals in Svirenko's (1915b) drawings: *P. caudatus* (fig. 8) and *P. tenuis* (figs. 17, 18) are so similar, that the differentiation of the two species is almost impossible. figure 17 has been indicated as the lectotype, as it is in this drawing that the cell has the most oval shape and has the lowest crest.

***Phacus tortus*** (Lemmermann) Skvortsov 1928: 110, pl.2, figs. 9, 10.

*Epitype:* Łukomska‐Kowalczyk et al. 2015, fig. 3n

*Representative DNA sequence:* GenBank KP944112

***Phacus triqueter*** (Ehrenberg) Dujardin 1841: 338

Cells oval (37–68 × 30–45 µm) with a high crest, triangular when cross‐sectioned; terminate with a straight or diagonal tail (Pochmann 1942; ).

*Representative DNA sequence*: SAG 1261‐8

*Representative strain:* GenBank AJ532485

***Phacus viridiozyra*** Kim & Shin 2014: 956, fig. 2H

*Type:* (see Kim and Shin 2014), permanently preserved material from the strain Sondang060709L deposited at the Chungnam National University, as number CNU 025431. Figure 2H in Kim and Shin 2014 shows the type.

*Representative DNA sequence:* GenBank KF744098

## DISCUSSION

On the oldest molecular phylogenetic trees, *Phacus* was recognized as a monophyletic member of the family Phacaceae (Kim et al. 2010), but further investigation suggested that the genus was paraphyletic (Kim and Shin 2014, Karnkowska et al. 2015). In those two studies *Phacus limnophilus* and *Phacus arnoldii (=P. warszewiczii*; Karnkowska et al. 2015) or only *P. limnophilus* (Kim and Shin 2014) sequences formed a sister clade to the clade grouping other sequences of *Phacus* and *Lepocinclis*. Later, the increased number of species and utilization of multi‐marker analyses confirmed the monophyly of *Phacus* (Kim et al. 2015). Our research, based on nSSU rDNA only, but including more sequences and *Phacus* taxa (136 sequences, 50 species), yielded a phylogenetic tree with a topology similar to those published previously. Most clades present on our tree consisted of sequences that were also together on previous trees. This includes our clade D (*P. oscillans* group), that underwent taxonomic revisions earlier (Karnkowska‐Ishikawa et al. 2010, Kim and Shin 2014) and is always strongly supported (Kim et al. 2010, 2015), clade J with sequences of *P. orbicularis* and *P. paraorbicularis* (Kim et al. 2010, 2015, Kim and Shin 2014) and clade C (*P. longicauda* and *P. helikoides* group; Kim and Shin 2014, Kim et al. 2015, Łukomska‐Kowalczyk et al. 2015) that was extended by adding sequences of *P. elegans*. Clades F, B, E, and H were also present on previous trees (Kim and Shin 2014, Kim et al. 2015), but herein they were verified and new sequences were added.

Clade I (*Phacus lismorensis*) is represented for the first time, clade G (*P. salinus*) was previously represented only by one sequence, and clade K was represented at most by three sequences of only one species (Łukomska‐Kowalczyk et al. 2015), now includes sequences of four species.

In the following part, the details concerning nomenclature and history of particular species and groups of species are discussed.

### **Phacus acuminatus**

Over 30 intraspecific taxa (subspecies, forms, and varieties) have been described for *Phacus acuminatus* (see: http://www.algaebase.org), mainly based on cell shape and size, tail length and shape as well as the number and morphology of the large paramylon grains. Many authors view such differences as intraspecific variability arising from either ontogenesis or environmental conditions (Huber‐Pestalozzi 1955, Popova and Safonova 1976 among others). This is further confirmed by our research. In regard to the shape and size of the cells, the obtained results (on average 30 × 20 µm) were compatible with the rich literature (e.g., Popova and Safonova 1976: 25–33 × 19–22 µm; short tail, no fold; Table 1) and the observed differences in each strain were the result of ontogenesis – the individuals tended to enlarge (particularly their width) right before cell division. Some dissimilarities were also found between strains, but they had no DNA sequence support – all strains occurred in a subclade of clade B (Fig. 2) with nSSU rDNA diversity below 3%. The UTEX 1317 strain (in the collection as *P. brachykentron*; Fig. 3a) was located among them. This morphological form, described by Pochmann (1942) as *P. brachykentron*, had been distinguished due to its slight cell asymmetry. For the aforementioned reasons, many taxa described in the literature as having a morphology similar to *P. acuminatus* and without proper diagnostic traits in their original descriptions, have been placed in synonymy.

### Phacus alatus, P. anomalus, and P. curvicauda

These three species are closely related (clade K in Fig. 2) and morphologically similar. They all possess thick cells (not flat) divided into two parts by a furrow – the halves are also twisted in the longitudinal axis. *Phacus alatus* was the first to be described (Klebs 1883), when Klebs came to the conclusion that the morphological form identified by Stein (1878) as *P. triqueter* (pl. 19, figs. 55–57) represented a species new to science. In the original diagnosis, Klebs drew attention to the atypical, characteristic shape (“widened, wing–like sides of the cell which overlap; one from the ventral side, the other from the dorsal”) and the lateral placement (“in both wings”) of two large, discoidal paramylon grains. Since then, many taxa had been described at various taxonomical ranks (species, varieties and forms) of a similar morphology, the proper identification of which is practically impossible. It even came to the point, where Pochmann (1942) described *P. platyaulax* for the same exact morphological form previously described by Klebs as *P. alatus*, i.e., *P. triqueter* from Stein's original work (1878; see p. 165 in Pochmann 1942). As a result, based on the aforementioned reasons and the research presented herein, many of those forms have been placed in synonymy under *P. alatus*.

Two more species were described in the 20^th^ century (*Phacus curvicauda* and *P. anomalus*) that differ from *P. alatus* by having spherical large paramylon grains positioned in the center of the cell (not parietal). As the criteria for their distinguishment were ambiguous, researchers tended to interpret their taxonomic rank in various ways (e.g., see Pochmann 1942; Popova and Safonova 1976, Starmach 1983). The species can be easily morphologically identified (Fig. 3, e–j); on the phylogenetic tree there is a clade with sequences of *P. anacoelus* sister to *P. anomalus*. Thus, distinguishing three separate taxa at the rank of species is justified. The shape and placement of the large paramylon grains are decent diagnostic traits that allow correct identification.

All three species are considered common and cosmopolitan (e.g., Popova and Safonova 1976, Starmach 1983). In Poland, *Phacus curvicauda* and *P. anomalus* are commonplace and are often found in large densities. *Phacus alatus*, on the other hand, is found rarely and only in low densities (B. Zakryś, pers. obs.). One other species – *P. drezepolski* (Stawiński 1969) had been described from Poland based on a single cell – however, as it is not different from *P. anomalus*, it has been listed as one of its synonyms.

### Phacus anacoelus

This euglenid of a particularly characteristic shape (almost spherical, slightly flattened, with 4 spiral ribs; Fig. 3, l–o) was described from the USA at the end of the 19^th^ century by Stokes (1885b). Forty years later, Sokoloff (1933) described a similar species from ponds in Mexico, but due to the asymmetry of the cell the species was named *P. asymmetricus*. Jahn and Shawhan (1942) described *P. quinquemarginatus* with 5 ribs from canals and ponds in Iowa (USA). All of the aforementioned species are of a similar size (on average around 40 × 35 µm; Table 1). Some authors of critical monographs recognized only *P. anacoelus*, deeming it a very rare species (Pochmann 1942; Popova and Safonova 1976). *Phacus quinquemarginatus* and *P. asymmetricus* had been reported from Argentina (Tell and Conforti 1986), while *P. anacoelus* had been recorded in Russia (Popova and Safonova 1976), Slovakia (Wołowski and Hindák 2005) and Poland (Dreżepolski 1925). We found two populations in Mazovia (Regnów and Izdebno Kościelne 1; see Fig. 1 and Table S1), one of which, in a small field pond in Izdebno Kościelne village, has been persisting for the past several years and appears regularly during summertime in high densities. The Polish populations have cells with bilateral symmetry visible in a cross section, but less so when observing mobile cells (Fig. 3, l–o). Thus, the term “asymmetrical” might be interpreted in various ways, and there is no basis to distinguish these species based on their imprecise, original diagnoses. However, we continue to recognize these three taxa pending the study of additional material. The sequences of *P. anacoelus* are located on the phylogenetic trees in a subclade within clade K (Fig. 2) along with *P. curvicauda, P. anomalus* and *P. alatus*, despite that “at first glance” these species appear morphologically very dissimilar (Fig. 3, e–j, l–o). However, they have a similar cell shape: slightly flattened, with well‐developed ventral and dorsal sides, terminating with a sharp, hyaline tail bent toward the dorsal side.

### Phacus ankylonoton

This morphological form was described from Poland by Dreżepolski (1925) as *Phacus caudata* var. *polonica*, and later was recognized as separate species (*P. ankylonoton*) by Pochmann (1942). It differs from *P. caudatus* in the width of the cell, which in cross‐section is triangular (Fig. 3q, Table 1). The validity of distinguishing this species is further confirmed by two DNA sequences (strain CCAC 0043 from Germany and Mokpo033107E from South Korea), creating a common clade (H) on the phylogenetic tree at a distant position from the sequences of *P. caudatus* (clade E in Fig. 2). The species is rare – it has been noted only a few times in Europe, Asia and South America (Shi et al. 1999, Popova and Safonova 1976, Starmach 1983, Tell and Conforti 1986). In Poland it was previously reported (Stawiński 1969, Wołowski 1998), but we never found it ourselves.

### Phacus applanatus

Two strains, CCAC 2604B (=ASW 08023) isolated in the Netherlands (channel in Leiden) and Beopsu030709E isolated in South Korea, occurred in clade H (Fig. 2 and Table S1). The morphological study of the CCAC 2604B strain demonstrates that it is an elongated‐oval, flat cell that terminates with a straight tail (Fig. 3r). These are the only reports of this species across the world. It is yet to be found in Poland.

### Phacus arnoldii and P. limnophilus

These two taxa represent the basal branch of the *Phacus* phylogenetic tree (clade A in Fig. 2), despite being morphologically “more similar” to *Lepocinclis*. The greatest similarity is the three‐ridged cell shape of *P. arnoldii*, alike *L. tripteris,* while the spindle‐shaped (unflattened) cells of *P. limnophilus* are typical of the majority of *Lepocinclis* species (Fig. 4, b, c and g‐i). Increasing the number of DNA sequences only further supported this positioning. *Phacus arnoldii* had been described from Ukraine in 1915, but its presence is often reported in the literature as *P. warszewiczii* Drezepolski; named after Poland's capital city, Warsaw (“Warszawa” in Polish). In Poland *P. arnoldii* occurs rarely and always in small densities, in contrast to *P. limnophilus*, which is much more common and may create dense populations (B. Zakryś, pers. obs.). Both species are considered cosmopolitan (e.g., Popova and Safonova 1976, Starmach 1983, Tell and Conforti 1986, Shi et al. 1999).

The variety described from Russia (*Phacus arnoldii* var. *ovatus*; Popova 1947) has been included as a synonym of *P. arnoldii*, as minute differences in cell shape are not diagnostic – the sequence of the Polish strain (one with more rounded cells) is sister to a strain isolated in Austria (CCAC 2432E, =ASW 08064), the shape of which (less round, more elongated) seems to be a result of culturing conditions.

### Phacus caudatus and P. manginii

These two species are closely related and morphologically similar due to their well‐developed (tall) dorsal fold that elongates into a sharp, hyaline, slightly ventrally bent tail, but they do differ in cell shape and size (subclades in clade E in Fig. 2; Fig. 3, s–u, Table 1). *Phacus caudatus* was described at the end of 19^th^ century (Hübner 1886). Later, several varieties were distinguished (see http://www.algaebase.org) based on minute cell shape and size differences. We did not confirm the validity of several of these forms, which is why they are being synonymized under *P. caudatus*. The presence of Austrian, Korean, Romanian and Polish strains in the *P. caudatus* clade affirms the cosmopolitan nature of this species. Daepyeong101908G, as well as ASW 08020 (=CCAC 2415 B) strain, identified as *P. carinatus* in previous works, and likewise Suwol060709C strain, identified as *P. swirenkoi* (Kim and Shin 2014, Kim et al. 2015), have been placed in synonymy under *P. caudatus* based on their placement in the *P. caudatus* clade as well as their morphology (Table 1, Fig. 3, s and t). Moreover *P. carinatus* is a very dubious species. This morphological form was first identified in Australia by Playfair (1921, as *P. triqueter*) and later considered a new species (*P. carinatus*) by Pochmann (1942). It has not been found since. Moreover it is not present in any critical monograph on euglenids (Popova and Safonova 1976, Tell and Conforti 1986, Shi et al. 1999).

In Poland, *Phacus caudatus* is common and occasionally can be found in dense populations, similarly to *P. manginii* described from Indochina (Léfévre 1931). The latter has rarely been mentioned in the literature (from China, Shi et al. 1999; and Argentina, Tell and Conforti 1986 among others), most likely due to being mistaken for *P. caudatus*. However, its presence in Europe does affirm its cosmopolitan status. The Gungnamji052507C strain (identified as *P. triqueter*) is renamed as *P. manginii* due to the position of its sequence in the *P. manginii* clade.

The variety described by Dreżepolski from Poland as *Phacus caudatus* var. *polonica* was recognized by Pochmann (1942) as separate species (*P. ankylonoton*), which has been supported by this research – the sequence of CCAC 0043 strain (mistakenly identified in the collection as *P. ranula*) occurred in a clade with the Korean strain located far from the *P. caudatus* clade.

### Phacus elegans and P. lismorensis

According to Pochmann these two very characteristic, yet still very similar morphological forms represent two species – *Phacus elegans* can be distinguished from *P. lismorensis* based on "a more elongated cell form and a shorter tail” (Pochmann 1942: 199). The Polish strains representing both morphological forms occur in different clades, *Phacus elegans* close to species with long tails (*P. longicauda, P. helikoides* among others), whereas *P. lismorensis* occurs in clade I in Figures 2 and S1. Furthermore, we have established that cell shape is a diagnostic morphological feature – with a bow–like arch and a visibly ventrally bent tail in *P. lismorensis* and straight (elongated) in *P. elegans* (Fig. 4, a and n). Additionally, *P. elegans* has a low crest that causes it to be slightly thicker than *P. lismorensis*. In Poland both species occur rarely and in minute densities. In the literature *P. lismorensis* is mentioned often and is considered to be cosmopolitan (e.g., Popova and Safonova 1976, Starmach 1983, Tell and Conforti 1986, Shi et al. 1999). On the other hand, *P. elegans* is rarely noted, which might be the result of the doubt over the validity of distinguishing the two species (Popova and Safonova 1976, Tell and Conforti 1986).

### Phacus gigas and P. hamatus

These two species occur in the same clade (F in Fig. 2), despite being morphologically different both in cell shape and size – *Phacus gigas* is flat, slightly asymmetrical and large (the largest of all species in *Phacus* – on average 100 × 75 µm), whereas *P. hamatus* has symmetrical, much smaller (on average 50 × 30 µm) and spoon‐shaped (convex) cells (Table 1, Fig. 4, j and k). Despite such characteristic features, until recently some researchers questioned the validity of distinguishing *P. gigas* (e.g., Popova 1947, Popova and Safonova 1976). However both Bennett and Triemer (2012) and herein, in which the number of sequences for both species has been increased, unambiguously support two distinct species with interspecific nSSU rDNA variability above 7%. *Phacus hamatus* (as *P. pleuronectes* var. *citriformis*) has been described from Poland (Dreżepolski 1922), while *P. gigas* from Brazil (Cuncha 1913). Both species are cosmopolitan (Starmach 1983, Tell and Conforti 1986, Shi et al. 1999). In Poland *P. hamatus* is common, whereas *P. gigas* is far more rare, however both may occur in large densities (B. Zakryś, pers. obs.).

### Phacus granum

This species had been described from Poland (Dreżepolski 1925) based on the presence of large paramylon grains in the cell that in the light of current knowledge is not a diagnostic trait, as it depends on the physiological state of the organism. Sadly, due to the small cell size and its morphological similarity to other representatives of the “small *Phacus*” group (*P. brevisulcus, P. claviformis, P. hordeiformis, P. longisulcus, P. minimus, P. oscillans, P. parvulus, P. polytrophis, P. pusillus,* and *P. skujae –* clade D in both Figs. 2; S1), we have failed to isolate material from environmental samples. Most likely, based on the same reasons, it is often omitted in the literature, despite being reported as cosmopolitan by some authors (Popova and Safonova 1976, Starmach 1983, Tell and Conforti 1986).

### Phacus minutus

This morphological form has been described from Australia (a botanical garden in Sydney) as a variety of *Phacus pleuronectes* (*P. pleuronectes* var. *minuta*; Playfair 1921) due to their similar, ovoid cell shape, but of a smaller size (*P. pleuronectes*: 39–55 × 22–33 µm; *P. minutus*: 20–30 × 11–25 µm). Later, it was raised to the rank of species by Pochmann (1942). In his diagnosis, Pochmann stressed additional differences from *P. pleuronectes*, such as a more flattened cell shape. The validity of distinguishing this taxon is confirmed by our research. Although two sequences (Korean and Portuguese) form a sister clade with sequences of *P. pleuronectes* (Fig. 2, clade B subclades), the sequence divergence (2.4–4%) and morphological features (cell size and shape) allowed us to decide that *P. pleuronectes* and *P. minutus* should be accepted as distinct species (Fig. 3, c, d and p), at least pending the inclusion of new strains morphologically similar to *P. minutus* in the analyses. Moreover cells of the Portuguese strain (ACOI 1755) correspond morphologically to the original description by Pochmann with the exception of the tail, which is bent toward the dorsal side rather than sideways.

### Phacus orbicularis and P. paraorbicularis

The two common, cosmopolitan and morphologically similar species are sister taxa (sequence divergence 0.7–1.4 %) and have already been verified taxonomically (Kosmala et al. 2007, Kim and Shin 2014). *Phacus orbicularis* was described in the 19th century (Hübner 1886), whereas *P. paraorbicularis* was described recently based on morphological and DNA sequence data (Kim and Shin 2014). In Poland, *P. paraorbicularis* occurs commonly and often in high densities. *Phacus orbicularis* is far rarer, and its populations are much more varied in cell shape and size (Kosmala et al. 2007). The form we collected in Poland is often known in the literature as *P. heimii* (symmetrical cells [31 × 23 µm] with a short tail [4 µm]; Fig. 3k). Its SSU sequence (MN149583 of the isolate UW2362Choc) places it in the *P. orbicularis* clade, confirming that *P. heimii* is a synonym of *P. orbicularis* (subclade in clade J in Figs. 2, S1).

### Phacus pleuronectes

This species was taxonomically verified (Kosmala et al. 2007). After the addition of new sequences, it remains in a well‐established clade, in which two groups can be distinguished – one consists of the sequences from culture strains and one from Poland, while the other groups Polish strains only (Fig. S1). The cells from the Polish populations have a slightly more trapezoid–like shape and are slightly smaller (30–37 × 20–25 µm; Fig. 3p, Table 1) in comparison with cultured cells (38–55 × 26–28 µm – see table 2 in Kosmala et al. 2007). As genetic diversity in the clade is low, those morphological differences might be a result of different living conditions. The trapezoid–like form had been described from Poland under the name of *P. trapezoides* (Stawiński 1969) and from China as *P. trapezialis* (Shi 1987). Both names are now included in the list of synonyms for *P. pleuronectes*. It is cosmopolitan and a common species in Poland.

### Phacus raciborskii

The species of *Phacus* with flat and permanently spirally twisted cells are located in different clades (see *P. inflexus, P. smulkowskianus, P. tortus* or *P. helikoides* – Figs. 2, S1). Another taxon of such morphology is *P. raciborskii*, described from Poland (Dreżepolski 1925). Four sequences of Polish strains have joined the Portuguese (strain ACOI 1758), two Korean (Gungnamji052507B as *P. trimarginatus* and Leynes012810F as *P. mariae* in Kim and Shin 2014, Kim et al. 2015) and the ASW 08004 (as *Phacus* sp.) strain sequences, creating a well‐supported clade (Fig. 2, clade E subclade, Fig. S1). The Portuguese sequence had been verified based on images posted on the website of the ACOI culture collection (http://acoi.ci.uc.pt/spec_detail.php?cult_id=1442). In the literature, we have found one more species (*P. trimarginatus*), described from ponds in Iowa (USA), that is morphologically indistinguishable from *P. raciborskii* and therefore has been included as a synonym. *Phacus raciborskii* is widely accepted as cosmopolitan and common (Pochmann 1942, Popova and Safonova 1976, Starmach 1983). It had been noted in Poland for many years (Dreżepolski 1925, Czosnowski 1948, Stawiński 1969) and is often found in dense populations (B. Zakryś, pers. obs.).

### Phacus salinus

This very characteristic morphological form had been appearing in the literature since the 19^th^ century – first under the name *Crumenula texta* (Dujardin 1836: 204, pl. 9, fig. M), later as *Euglena texta* (Hübner 1886) or *Lepocinclis texta* (Lemmermann 1901). None of the aforementioned descriptions refer to the striation direction. Only Fritsch (1914) drew attention to this feature and described a species new to science (*L. salina*) due to its periplast striation (striae running from right to left). Popova (1955) however considered it to be a variant of *L. texta* (*L. texta* var. *salina*). Unexpectedly, based on DNA sequence data, *L. salina* is transferred to *Phacus* (*P. salinus*), in spite of being morphologically (oblong, rigid cells ; Fig. 4e) more similar to *Lepocinclis* (Linton et al. 2010). Until now only one sequence had been available (EU624028 of the strain of SAG 1244‐3), and its position was not well supported (Linton et al. 2010). Five additional sequences form a common, strongly supported clade with the previous sequence, but the position remains uncertain (clade G in Fig. 2, Fig. S1). *Phacus salinus* occurs commonly in Poland, often in large densities. Both forms (*texta* and *salina*) are universally accepted as cosmopolitan and common (e.g., Popova and Safonova 1976, Starmach 1983, Tell and Conforti 1986, Shi et al. 1999). In spite of that, we have never found *texta* (with periplast striation running from left to right), despite having inspected over 100 populations.

Variability of nSSU rDNA fragments (lengths and sequences) of *Phacus salinus* is much higher than the observed morphological diversity. There are also no morphological features that could be used to distinguish the species. This occurs in several common euglenid species with very high intraspecific genetic diversity, e.g., *Lepocinclis fusiformis* up to 7.8%, *L. hispidulus* up to 5.1% (Łukomska‐Kowalczyk et al. 2020), and *P. circumflexus* up to 4.9% (Łukomska‐Kowalczyk et al. 2015).

The sequence MN149599 of *Phacus salinus* UW1948Wos (3,560 bp) is almost the longest known nSSU rDNA in autotrophic euglenids, only that of *Euglena sanguinea* is longer‐the fragment exceeds 6,000 bp (Karnkowska‐Ishikawa et al. 2013).

### Phacus stokesii *and* P. segretii

Despite the two species being very similar morpologically (wide‐oval cells without a tail and with a long apical furrow (Fig. 4d and the photo of *Phacus segretii* strain ACOI 1337 on the collection's website: http://acoi.ci.uc.pt/spec_detail.php?cult_id=815), their sequences are found in separate clades (in clade H and as a sister to clade K in Fig. 2, Fig. S1). The most significant difference is cell size – *P. stokesii* is almost twice as big (40‐46 × 30‐35 µm) as *P. segretii* (22–28 × 20–22 µm; Table 1). In the literature, there are several other species of a similar morphology described, out of which five (*P. fominii*,* P. aspidion*,* P. balatonicus*,* P. starmachii,* and *P. betkowski*) are herein designated as synonyms of *P. stokesii*; one other (*P. stokesii* f. *minor*) has been synonymized under *P. segretii* due to a similar size and a lack of any other well‐defining feature. Popova and Safonova (1976: 62‐63) listed the same synonyms, except for *P. fominii*. Both species (*P. stokesii* and *P. segretii*) are considered cosmopolitan, but *P. stokesii* is far more common (Popova and Safonova 1976, Starmach 1983, Tell and Conforti 1986, Shi et al. 1999). Historically, only *P. stokesii* has been reported in Poland (Dreżepolski 1925, Stawiński 1969, Wołowski 1998), which currently is very uncommon and never found in large densities (B. Zakryś, personal observation)

### Phacus tenuis

The Portuguese strain ACOI 1757 (as *Phacus caudatus* in ACOI) occurs outside of the “*caudatus*” clade (clade H in Fig. 2), despite being morphologically very similar to *P. caudatus* (cells flat, elongated‐oval in shape; Figs. 3, s and t, 4f). It does however differ in terms of having a lower crest and a straight tail, which in turn makes it similar to *P. applanatus* (Fig. 3r). A detailed literature study shows, that such a morphological form was described from Ukraine by Svirenko (1915a), first as *P. caudatus* var. *tenuis*, and in the same year was raised to the rank of species (*P. tenuis*) by Svirenko (1915b). The research presented herein supports such a decision. *Phacus caudatus* var. *tenuis* is reported from various countries: Russia (Popova and Safonova 1976), the Czech Republic (Wołowski 1992), Slovakia (Wołowski and Hindák 1996) and Poland (Stawiński 1969).

### Phacus triqueter

The diagnostic trait for this species is its high crest that causes the cell's triangular appearance when cross‐sectioned. This three‐ridged cell shape of *Phacus triqueter* makes it similar to *Lepocinclis tripteris* or *P. arnoldii*. The very characteristic morphological form was described by Ehrenberg (1838) as *Euglena triquetra* and later moved to *Phacus* as *P. triqueter* (Dujardin 1841). Many authors considered it to be cosmopolitan (e.g., Pochmann 1942, Popova and Safonova 1976, Starmach 1983, Tell and Conforti 1986, Shi et al. 1999). Meanwhile, it has yet to be found in Poland by us, despite previous reports of its presence (Czosnowski 1948, Stawiński 1969, Wołowski 1998). Only one sequence of *P. triqueter* (from strain SAG 1261‐8) was included in the phylogenetic analysis (Fig. 2). Based on the image posted on the Culture Collection of Algae and Protozoa (CCAP) website, it appears that the strain had been verified correctly (https://www.ccap.ac.uk/results2014.php?mode=attr&Environment=All&Country=All&Pathogen=All&Type_Culture=All&Genus_Name=Phacus&Strain_Name=Phacus+triqueter&Strain_No=1261%2F8).

## conclusion

As a result of the presented studies the phylogenetic trees of *Phacus* now includes 50 species represented by 129 sequences of SSU rDNA, of which seven species (*Phacus anacoelus, P. anomalus, P. curvicauda, P. elegans, P. lismorensis, P. minutus,* and *P. stokesii*) and 55 sequences are new for the tree. The new sequences were obtained from laboratory strains (6) and those isolated directly from the environment (49). The environmental isolates came from 37 eutrophic water bodies located in Poland and one located in the Czech Republic. Comparative morphological and molecular studies on new laboratory strains and environmental isolates as well as research in the literature, have allowed (a) an estimation of morphological and genetic diversity and verification of morphological diagnostic features for particular taxa (well‐established clades on a molecular phylogenetic tree) and as a result their proper identification; due to the lack of morphological data, the taxonomic affiliation of five strains (Jigok090112 as *P. ranula* on the phylogeny tree; Leynes012810F as *P. mariae*; Burni081809, ASW 08004 and ACOI1138 as *Phacus* sp.) was not verified; (b) the reconstruction of phylogenetic relationships among 50 species of *Phacus* (c) taxonomic verification, emending diagnoses, and designating epitypes for well‐distinguished taxa (19 species).

## Supporting information


**Figure S1.** The Maximum Likelihood phylogenetic tree based on 136 nSSU rDNA sequences (of which 129 represent *Phacus*). The Bayesian posterior probability (pp) and the bootstrap (bs) values obtained by maximum likelihood analysis are marked at the nodes. The pp <0.75, bs values <50 and clades not present in the particular analysis are marked with a hyphen (‐). The sequences obtained in this study are indicated in bold type. Scale bar represents number of substitutions per site.

**Table S1.** List of species and sampling data of isolates/strains used in this study. GenBank accession numbers and their nuclear SSU rDNA gene sequenced are given, with new sequences indicated in bold type.

**Table S2.** The nuclear SSU rDNA pair‐wise sequence distances (%) among strains and isolates.
