# Supplementary figures and images for: Taxon‐rich phylogeny and taxonomy of the genus Phacus (Euglenida) based on morphological and molecular data
Source: J Phycol. 2020 Jun 26;56(5):1135–56. doi: 10.1111/jpy.13028 (PMC7687149; doi:10.1111/jpy.13028)

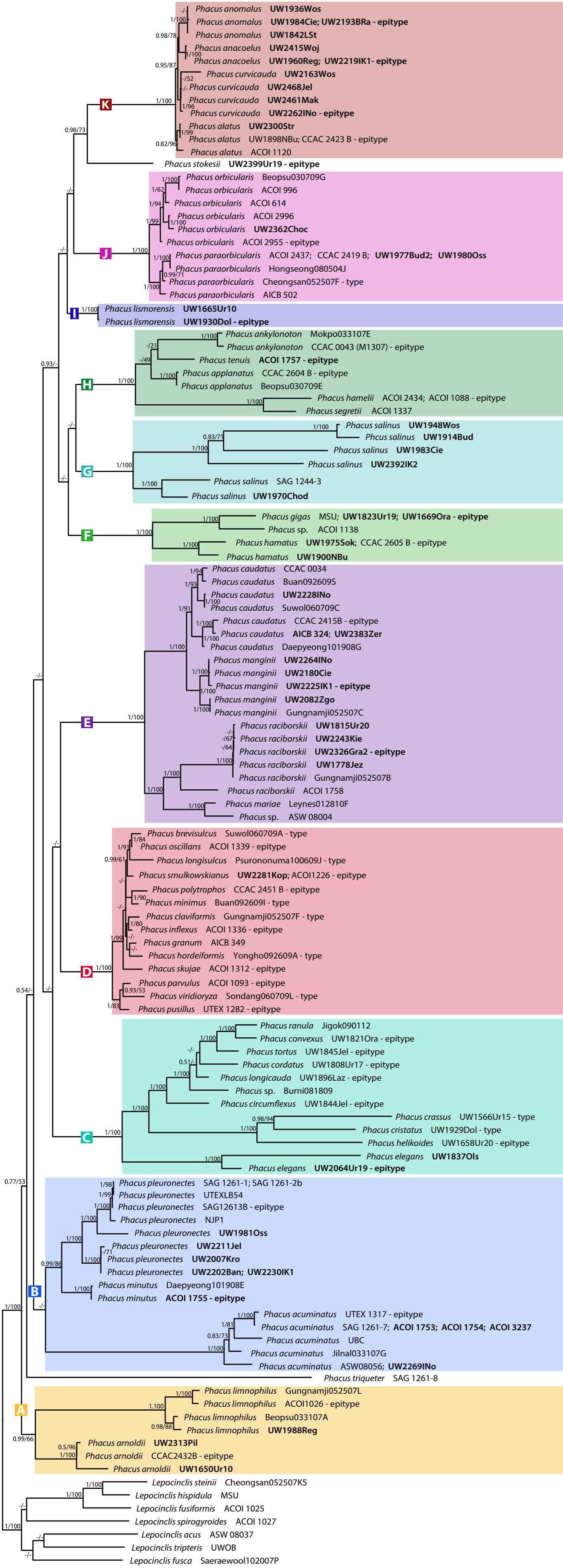

0.07

Supplement: Supplementary file 1 — Figure S1. The Maximum Likelihood phylogenetic tree based on 136 nSSU rDNA sequences (of which 129 represent Phacus). The Bayesian posterior probability (pp) and the bootstrap (bs) values obtained by maximum likelihood analysis are marked at the nodes. The pp <0.75, bs values <50 and clades not present in the particular analysis are marked with a hyphen (‐). The sequences obtained in this study are indicated in bold type. Scale bar represents number of substitutions per site. [file JPY-56-1135-s001.pdf]
