# Supplementary material for: Taxon‐rich phylogeny and taxonomy of the genus Phacus (Euglenida) based on morphological and molecular data
Source: J Phycol. 2020 Jun 26;56(5):1135–56. doi: 10.1111/jpy.13028 (PMC7687149; doi:10.1111/jpy.13028)
Supplement: Supplementary file 2 — Table S1. List of species and sampling data of isolates/strains used in this study. GenBank accession numbers and their nuclear SSU rDNA gene sequenced are given, with new sequences indicated in bold type. [file JPY-56-1135-s002.docx]

Table S1. List of species and sampling data of isolates/strains used in this study. GenBank accession numbers and their nuclear SSU rDNA gene sequenced are given, with new sequences indicated in bold type.

| Taxon | Isolate/strain codes | Number of cells used for DNA isolation | Accession no. of nuclear SSU rDNA | Origin | GPS | |
| --- | --- | --- | --- | --- | --- | --- |
| *L. acus* (O.F.Müll.)  B.Marin & Melkonian | ASW 08037 | pellet | AJ532458 | Austria, Neusiedler See at Illmitz, reed belt lacuna |  |  |
| *L. fusca* (G.A.Klebs)  Kosmala & Zakryś | Saeraewool102007P | pellet | KT304830 | South Korea |  |  |
| *L. fusiformis*  (H.J.Carter) Lemmerm. | ACOI 1025 | pellet | AY935697 | Portugal, Paúl do Boquilobo |  |  |
| *L. hispidula*  (Eichw.) V.Daday | MSU | 4 | HQ287918 | USA, Michigan, East Lansing |  |  |
| *L. spirogyroides*  B.Marin & Melkonian | ACOI 1027 | pellet | AY935692 | Portugal, Paúl do Boquilobo |  |  |
| *L. steinii*  Lemmerm. | Cheongsan052507K5 | pellet | KT304841 | South Korea |  |  |
| *L. tripteris* (Dujardin)  B.Marin & Melkonian | UWOB (=CCAP 1224⁄45) | pellet | AY935696 | Poland, Warsaw, large puddle near the Botanical Garden (Ujazdowskie 4 street) |  |  |
|  | **ACOI 1753** | pellet | **MN149548** | Portugal, Paul da Tornada |  |  |
|  | **ACOI 1754** | pellet | **MN149549** | Portugal, Coimbra, Convento de Sta Clara |  |  |
|  | **ACOI 3237** | pellet | **MN149550** | Portugal, Cernache, Colégio da Imaculada Conceição, lake |  |  |
| *P. acuminatus*  A.Stokes | ASW 08056 | pellet | AF283312 |  |  |  |
|  | Jilnal033107G | pellet | FJ719626 | South Korea |  |  |
|  | SAG 1261-7  (as *P. brachykentron*) | pellet | AJ532481 | United Kingdom, mud from Thames River near Oxford |  |  |
|  | UBC | pellet | GQ422787 | Canada, Vancouver, University of British Columbia, pond |  |  |
|  | UTEX 1317  (as *P. brachykentron*) | pellet | AF286209 | United Kingdom, Oxford, Thames River, mud |  |  |
|  | **UW2269INo** | 40 | **MN149553** | Poland, Izdebno Nowe village, pond | 52°08'01.3''N | 20°32'52.9''E |
| *P. alatus*  G.A.Klebs | ACOI 1120 | pellet | KF744089 | Portugal, Castelo Branco, Penamacor |  |  |
|  | ASW 08027  (as *P. platyaulax*) | pellet | AJ532474 | Austria, Neusiedler See, Mörbisch |  |  |
|  | UW1898NBu | 60 | KP944113 | Poland, Nowa Bukówka village, large puddle | 52°01'19.8''N | 20°38'28.3''E |
|  | **UW2300Str** | 50 | **MN149554** | Poland, Strzebielinek village, ditch | 54°42'2.96"N | 18°3'53.80"E |
| *P. anacoelus*  A.Stokes | **UW1960Reg** | 55 | **MN149555** | Poland, Regnów village, field pond | 51°44'58.9"N | 20°23'23.7"E |
|  | **UW2219IK1** | 30 | **MN149556** | Poland, Izdebno Kościelne village, field pond | 52°08'21.0''N | 20°32'03.2''E |
|  | **UW2415Woj** | 80 | **MN149557** | Poland, Wojnowce village, small pond | 53°30'51.8''N | 23°38'04.4''E |
| *P. ankylonoton* Pochm. | CCAC 0043  (as *P. ranula)* | pellet | KF744064 | Germany, Heligoland, freshwater pond |  |  |
|  | Mokpo033107E | pellet | KF744065 | South Korea |  |  |
| *P. anomalus*  F.E.Fritsch & M.F.Rich | **UW1842LSt** | 45 | **MN149558** | Czech Republic, Lásenický stav, pond | 49°07'09.2"N | 14°96'56.7"E |
|  | **UW1936Wos** | 40 | **MN149559** | Poland, Woszczele village, pond | 53°52'01.6"N | 22°16'07.5"E |
|  | **UW1984Cie** | 50 | **MN149560** | Poland, Cielądz village, park pond | 51°42'50.9"N | 20°20'44.7"E |
|  | **UW2193BRa** | 30 | **MN149561** | Poland, Biała Rawska village, fish pond | 51°48'06.1''N | 20°27'28.7''E |
| *P. applanatus*  Pochm. | CCAC 2604 B  (=ASW 08023) | pellet | EU624031 | Netherlands, channel in Leiden |  |  |
|  | Beopsu030709E | pellet | KF744066 | South Korea |  |  |
| *P. arnoldii*  Svirenko | CCAC 2432 B  (=ASW 08064)  (as *P. warszewiczii*) | pellet | GQ422793 | Austria, ephemeral pool in Baumgarten an der March (Lower Austria) |  |  |
|  | **UW1650Ur10** | 15 | **MN149562** | Poland, Urwitałt village, pond no.10 | 53°49'09.5"N | 21°39'21.8"E |
|  | **UW2313Pil** | 40 | **MN149563** | Poland, Pilchy village, small forest ponds near Roś Lake | 53°40'33.7"N | 21°51'11.7"E |
| *P. brevisulcus*  J.I.Kim & W.Shin | Suwol060709A | pellet | KF744067 | South Korea |  |  |
| *P. caudatus*  Hübner | **AICB 324** | pellet | **MN326770** | Romania, Dej (Cluj District), freshwater pond |  |  |
|  | CCAC 2415 B  (=ASW 08020) | pellet | AJ532482 | Belgium, De Zegge |  |  |
|  | CCAC 0034 | pellet | KF744070 | Belgium, nature reserve De Zegge |  |  |
|  | Buan092609S | pellet | KF744069 | South Korea |  |  |
|  | Daepyeong101908G  (as *P. carinatus*) | pellet | KF744068 | South Korea |  |  |
|  | Suwol060709C  (as *P. swirenkoi*) | pellet | KF744094 | South Korea |  |  |
|  | **UW2228INo** | 30 | **MN149567** | Poland, Izdebno Nowe village, pond | 52°08'01.3''N | 20°32'52.9''E |
|  | **UW2383Zer** | 80-100 | **MN149565** | Poland, Warsaw, ditch with tip drainage | 52°18′24.1″N | 20°58′05.0″E |
| *P. circumflexus*  Pochm. | UW1844Jel | 40 | KP944083 | Poland, Jelonki (district of Warsaw), Schneider’s Pond | 52°13'05.6''N | 20°54'41.5''E |
| *P. claviformis*  J.I.Kim & W.Shin | Gungnamji052507F | pellet | KF744071 | South Korea |  |  |
| *P. convexus*  (Pochm.) Zakryś & M.Łukomska | UW1821Ora | 70 | KP944090 | Poland, Oracze village, farm pond | 53°52'36.6"N | 22°20'41.2"E |
| *P. cordatus*  (Pochm.) Zakryś | UW1808Ur17 | 30 | KP944102 | Poland, Urwitałt village, pond no. 17 | 53°50'48.1"N | 21°36'43.4"E |
| *P. crassus*  Zakryś & M.Łukomska | UW1566Ur15 | 30 | KP944108 | Poland, Urwitałt village, pond no. 15 | 53°49'97.8"N | 21°38'57.6"E |
| *P. cristatus*  Zakryś & M.Łukomska | UW1929Dol | 12 | KP944109 | Poland, Doliwy village, fish pond | 54°01'55.5''N | 22°18'02.0''E |
| *P. curvicauda*  Svirenko | **UW2163Wos** | 30 | **MN149566** | Poland, Woszczele village, pond | 53°52'01.6"N | 22°16'07.5"E |
|  | **UW2262INo** | 30 | **MN149567** | Poland, Izdebno Nowe village, pond | 52°08'01.3''N | 20°32'52.9''E |
|  | **UW2461Mak** | 70 | **MN149568** | Polska, Mąkolno, łowisko koło cmentarza | 52°21′32,4″N | 18°34′46,0″E |
|  | **UW2468Jel** | 60 | **MN149569** | Poland, Jelonki (district of Warsaw), Schneider’s Pond | 52°13'05.6'' N | 20°54'41.5''E |
| *P. elegans*  Pochm. | **UW1837Ols** | 10 | **MN149570** | Poland, Olsztyn town, pond | 53°45′05.6″N | 20°27′15.5″E |
|  | **UW2064Ur19** | 20 | **MN149571** | Poland, Urwitałt village, pond no. 19 | 53°50'38.0"N | 21°38'06.5"E |
| *P. gigas*  A.M.Cunha | MSU | 4 | HQ287922 | USA, Michigan, East Lansing |  |  |
|  | **UW1669Ora** | 20-30 | **MN149572** | Poland, Oracze village, farm pond | 53°52'36.6"N | 22°20'41.2"E |
|  | **UW1823Ur19** | 30 | **MN149573** | Poland, Urwitałt village, pond no. 19 | 53°50'38.0"N | 21°38'06.5"E |
| *P. granum*  Drezep. | AICB 349 | pellet | DQ249880 | Romania, Dej (Cluj District), freshwater pond |  |  |
| *P. hamatus*  Pochm. | CCAC 2605 B  (=ASW 08032, =M 2605) (as *P. pleuronectes*) | pellet | AJ532473 | Austria, bay of Rust, Seeterrasse, lake Neusiedlersee |  |  |
|  | **UW1900NBu** | 50 | **MN149574** | Poland, Nowa Bukówka village, pond | 52°01'19.1''N | 20°38'27.9''E |
|  | **UW1975Sok** | 50 | **MN149575** | Poland, Sokolec village, field pond | 53°03'18.8"N | 17°15'34.2"E |
| *P. hamelii*  P.Allorge & M.Lefèvre | ACOI 1088 | pellet | FJ719628 | Portugal, Ribeira de Nisa, Açude do Racheiro |  |  |
|  | ACOI 2434 | pellet | DQ397673 | Portugal |  |  |
| *P. helikoides*  Pochm. | UW1658Ur20 | 25 | KP944094 | Poland, Urwitałt village, pond no. 20 | 53°51'15.7"N | 21°36'57.6"E |
| *P. hordeiformis*  J.I.Kim & W.Shin | Yongho092609A | pellet | KF744073 | South Korea |  |  |
| *P. inflexus*  (I.Kisselev) Pochm. | ACOI 1336 | pellet | FJ590503 | Portugal, Paul da Tornada |  |  |
| *P. limnophilus*  (Lemmerm.) E.W**.**Linton & Karnk. | ACOI 1026 | pellet | DQ249877 | Portugal, Montemor-o-Velho, Sta Olaia |  |  |
|  | Beopsu033107A | pellet | KF744075 | South Korea |  |  |
|  | Gungnamji052507L | pellet | KF744077 | South Korea |  |  |
|  | **UW1988Reg** | 60 | **MN149576** | Poland, Regnów village, field pond | 51°44'58.9"N | 20°23'23.7"E |
| *P. lismorensis* Playfair | **UW1665Ur10** | 15 | **MN149577** | Poland, Urwitałt village, pond no.10 | 53°49'09.5"N | 21°39'21.8"E |
|  | **UW1930Dol** | 10 | **MN149578** | Poland, Doliwy village, fish pond | 54°01'55.5''N | 22°18'02.0''E |
| *P. longicauda*  (Ehrenb.) Dujard. | UW1896Laz | 60 | KP944103 | Poland, Łazienki Park in Warsaw, pond | 52°12'47.8''N | 21°01'48.7''E |
| *P. longisulcus*  J.I.Kim & W.Shin | Psurononuma100609J | pellet | KF744079 | Japan |  |  |
| *P. manginii*  M.Lefèvre | Gungnamji052507C  (as *P. triquetra*) | pellet | KF744096 | South Korea |  |  |
|  | **UW2082Zgo** | 30 | **MN149579** | Poland, Zgorzała Lake near Warsaw city | 52°07'17.9"N | 20°59'46.6"E |
|  | **UW2180Cie** | 30 | **MN149580** | Poland, Cielądz village, park pond | 51°42'50.96"N | 20°20'44.77"E |
|  | **UW2225IK1** | 30 | **MN149581** | Poland, Izdebno Kościelne village, field pond | 52°08'21.0''N | 20°32'03.2''E |
|  | **UW2264INo** | 25 | **MN149582** | Poland, Izdebno Nowe village, pond | 52°08'01.3''N | 20°32'52.9''E |
| *P. mariae*  Deflandre | Leynes012810F | pellet | KF744080 | Philippines |  |  |
| *P. minimus*  J.I.Kim & W.Shin | Buan092609I | pellet | KF744081 | South Korea |  |  |
| *P. minutus*  (Playfair) Pochm. | **ACOI 1755**  (as *P. acuminatus*) | pellet | **MN149551** | Portugal, Quiaios, Lagoa da Vela |  |  |
|  | Daepyeong101908E  (as *Phacus* sp.) | pellet | KF744088 | South Korea |  |  |
| *P. orbicularis*  Hübner | ACOI 2955 | pellet | DQ397671 | Portugal |  |  |
|  | ACOI 2996 | pellet | DQ397668 | Portugal |  |  |
|  | ACOI 614 | pellet | DQ397664 | Sintra, Parque de Monserrate, water tank |  |  |
|  | ACOI 996 | pellet | AJ532478 | Portugal, Barragem do Azibo |  |  |
|  | Beopsu030709G | pellet | KF744082 | South Korea |  |  |
|  | **UW2362Choc** | 45 | **MN149583** | Poland, Choczewo village, pond | 54°44'09.5"N | 17°52'51.2"E |
| *P. oscillans*  G.A.Klebs | ACOI 1339 | pellet | FJ590499 | Portugal, Paul da Tornada |  |  |
| *P. paraorbicularis*  J.I.Kim & W.Shin | ACOI 2437  (as *P. orbicularis*) | pellet | DQ397672 | Portugal |  |  |
|  | AICB 502  (as *P. orbicularis*) | pellet | AY935698 | Romania, Stejeris (Cluj District), freshwater pond |  |  |
|  | CCAC 2419 B  (=ASW 08054)  (as *P. orbicularis*) | pellet | AF283315 | Austria, Danubian backwater near Eckartsau Lower Austria |  |  |
|  | Cheongsan052507F | pellet | KF744083 | South Korea |  |  |
|  | Hongseong080504J | pellet | KF744085 | South Korea |  |  |
|  | **UW1977Bud2** | 50 | **MN149584** | Poland, Budzyń village, pond | 52°53'43.9"N | 16°59'45.9"E |
|  | **UW1980Oss** | 50 | **MN149585** | Poland, Ossowice village, fish pond | 51°43'34.2"N | 20°18'06.3"E |
| *P. parvulus*  G.A.Klebs | ACOI 1093 | pellet | AJ532472 | Portugal, Castelo Branco (Penha Garcia), pond |  |  |
| *P. pleuronectes*  (O.F.Müll.) Nitzsch | NJ P1 | pellet | FJ719633 | United States |  |  |
|  | SAG 1261-1  (=UTEX 1288)  (as *P. acuminatus*) | pellet | AJ532477 | United Kingdom, Cherry Hinton near Cambridge |  |  |
|  | SAG 1261-2b  (as *P. alatus*) | pellet | AJ532476 | United Kingdom, ditch in Trumpington |  |  |
|  | SAG 1261-3b | pellet | AJ532475 | United Kingdom |  |  |
|  | UTEX LB54 | pellet | GQ422791 |  |  |  |
|  | **UW1981Oss** | 40 | **MN149586** | Poland, Ossowice village, fish pond | 51°43'34.2"N | 20°18'06.3"E |
|  | **UW2007Kro** | 70 | **MN149587** | Poland, Kromnów village, small pond | 52°22'39.1"N | 20°21'08.1"E |
|  | **UW2202Ban** | 40 | **MN149588** | Poland, Baniocha village, field pond | 52°01'50.4"N | 21°07'30.5"E |
|  | **UW2211Jel** | 7 | **MN149589** | Poland, Jelonki (district of Warsaw), Schneider’s Pond | 52°13'05.6'' N | 20°54'41.5''E |
|  | **UW2230IK1** | 40 | **MN149590** | Poland, Izdebno Kościelne village, field pond | 52°08'21.0''N | 20°32'03.2''E |
| *P. polytrophos*  Pochm. | CCAC 2451 B  (=ASW 08018)  (as *P. oscillans*) | pellet | FJ590498 | Austria, reed channel near Breitenbrunn, lake Neusiedlersee |  |  |
| *P. pusillus*  Lemmerm. | UTEX 1282 | pellet | AF190815 |  |  |  |
| *P. raciborskii*  Drezep. | ACOI 1758 | pellet | FJ719634 | Portugal, Montemor-o-Velho, Paul do Taipal, canal |  |  |
|  | Gungnamji052507B  (as *P. trimarginatus*) | pellet | FJ719637 | South Korea |  |  |
|  | **UW1778Jez** | 30 | **MN149591** | Poland, Jeziorowskie village, bay of the Lake Sawinda Mała | 53°54'36.3''N | 22°12'48.6''E |
|  | **UW1815Ur20** | 30 | **MN149592** | Poland, Urwitałt village, pond no. 20 | 53°51'15.7"N | 21°36'57.6"E |
|  | **UW2243Kie** | 25 | **MN149593** | Poland, Kiełpińskie Lake | 52°21'41.9"N | 20°52'31.8"E |
|  | **UW2326Gra2** | 40 | **MN149594** | Poland, Grabówek village, field pond | 53°50'52.9"N | 21°42'19.6"E |
| *P. ranula*  Pochm. | Jigok090112 | pellet | KF744092 | South Korea |  |  |
| *P. salinus*  (F.E.Frtisch) E.W**.**Linton & Karnk. | SAG 1244-3 | pellet | EU624028 | United Kindom |  |  |
|  | **UW1914Bud** | 300 | **MN149596**  **MN149597** | Poland, Budzyń village, pond | 52°53'19.7''N | 16°59'05.0''E |
|  | **UW1948Wos** | 300 | **MN149599** | Poland, Woszczele village, pond | 53°52'01.6"N | 22°16'07.5"E |
|  | **UW1970Chod** | 50 | **MN149598** | Poland, Chodzież village, park pond | 52°59'42.7"N | 16°54'11.8"E |
|  | **UW1983Cie** | 300 | **MN149595** | Poland, Cielądz village, park pond | 51°42'50.9"N | 20°20'44.7"E |
|  | **UW2392IK2** | 50 | **MN149600** | Poland, Izdebno Kościelne village, country pond | 52°08'16,8''N | 20°31'52,9''E |
| *P. segretii*  P.Allorge & M.Lefèvre | ACOI 1337 | pellet | FJ719635 | Portugal, Paul da Tornada |  |  |
| *P. skujae*  Skvortsov | ACOI 1312 | pellet | FJ597146 | Portugal, Castelo Branco, pond near Zebreira |  |  |
| *P. smulkowskianus*  (Zakryś) Kusber | ACOI 1226 | pellet | DQ249881 | Portugal, Quiaios, Lagoa da Vela |  |  |
|  | **UW2281Kop** | 40 | **MN149601** | Poland, Kopalino village, small pond | 54°47'19.4"N | 17°51'04.9"E |
| *Phacus* sp. | ACOI 1138  (as *P. hamatus*) | pellet | KF744072 | Portugal, Paúl do Boquilobo |  |  |
|  | ASW 08004  (as *P. acuminatus*) | pellet | AF283311 |  |  |  |
|  | Burni081809  (as *P. longicauda*) | pellet | KF744078 | South Korea |  |  |
| *P. stokesii*  Lemmerm. | **UW2399Ur19** | 15 | **MN149602** | Poland, Urwitałt village, pond no. 19 | 53°50'38.0"N | 21°38'06.5"E |
| *P. tenuis*  Svirenko | **ACOI 1757**  (as *P. caudatus*) | pellet | **MN149552** | Portugal, Quiaios, Lagoa da Vela |  |  |
| *P. tortus*  (Lemmerm.) Skvortsov | UW1845Jel | 50 | KP944112 | Poland, Jelonki (district of Warsaw), Schneider’s Pond | 52°12'47.8''N | 21°01'48.7''E |
| *P. triqueter*  (Ehrenb.) Dujard. | SAG 1261-8 | pellet | AJ532485 | United Kingdom, Cambridge, Shelford |  |  |
| *P. viridioryza*  J.I.Kim & W.Shin | Sondang060709L | pellet | KF744098 | South Korea |  |  |

**ACOI**, Culture Collection of Algae at the Department of Botany, University of Coimbra, Portugal; **AICB**, Culture Collection of Algae at the Institute of Biological Research Cluj-Napoca, Romania; **ASW**, Culture Collection of Algae at the University of Vienna, now available from CCAC; **CCAC**, Culture Collection of Algae, University of Cologne, Germany; **SAG**, Sammlung von Algenkulturen Pflanzenphysiologisches Institut der Universität Göttingen, Germany; **UTEX**, Culture Collection of Algae at the University of Texas at Austin, TX, USA; **UW**, University of Warsaw.
